# Wake Up and Smell the Infected Bees: Volatile Cues of *Vairimorpha* Infection in Honey Bees

**DOI:** 10.1002/ece3.73768

**Published:** 2026-06-04

**Authors:** Ayman Asiri, Sarah E. Perkins, Carsten T. Müller

**Affiliations:** ^1^ School of Biosciences Cardiff University Cardiff UK; ^2^ Game & Wildlife Conservation Trust The Allerton Project Loddington UK

**Keywords:** chemical cues, chemical signalling, pathogen surveillance, social insects, volatile organic compounds

## Abstract

Infection can alter volatile organic compounds (VOCs) across a wide range of taxa, but the effects in adult honey bees remain unexplored. Adult workers are major contributors to colony odour and play a central role in both pathogen transmission and collective disease defence (social immunity). We characterised VOC emissions from *Vairimorpha*‐infected and uninfected worker honey bees over a 14‐day infection time series using dynamic headspace sampling and two‐dimensional gas chromatography coupled with mass spectrometry (GC × GC–MS). *Vairimorpha* spp. are globally important gut parasites linked to colony declines. VOC profiles differed significantly between control and infected bees when pooled across all time points, and at days 6 and 12 post‐infection, suggesting a ‘smell’ of infection. Random forest and redundancy analysis identified tetradecane, dodecane, and one unidentified compound as characteristic of early‐stage infections, and 3,4‐dimethylbenzaldehyde as more abundant during late‐stage infections. Our findings provide the first evidence that *Vairimorpha* spp. infection alters the VOC profile of adult honey bees. We detected robust shifts in VOC profiles that colonies may use to detect disease, which could be harnessed for applied surveillance and/or highlight a potential role for infection odours in regulating social immunity, offering new directions for understanding how parasites interact with host communication systems in social animals.

## Graphical abstract

Infection can modify host volatile emissions, yet effects in adult honey bees remain unexplored. Using dynamic headspace sampling and GC × GC–MS, we show that *Vairimorpha* spp. infection significantly alters the volatile profile of adult worker honey bees over a 14‐day time series, producing stage‐specific shifts in multivariate odour structure. These findings provide the first evidence that adult infection reshapes honey bee volatile chemical phenotype and suggest a potential role for infection odours in social immunity and disease surveillance.

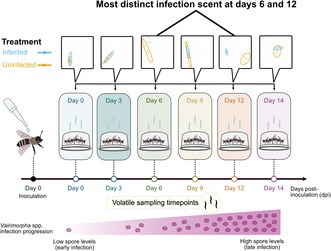

## 1 Introduction

Chemical sensing is an ancient sense, shared across all forms of life, from bacteria to animals (Liberles 2014; Wyatt 2014; Blomquist et al. 2020). Crucially, chemical sensing may also play a role in disease, because infection can alter volatile organic compounds (VOCs). Infection‐linked shifts in VOCs occur in humans (Shirasu and Touhara 2011; Ahmed et al. 2017), mice (Ehman and Scott 2001) and cattle (Peled et al. 2012) and can serve as distinctive biomarkers of infection (Calcagnile et al. 2019; Gaude et al. 2019). For social animal species, infection‐associated chemical cues may inform healthy individuals to avoid diseased conspecifics, thereby reducing transmission (Kiesecker et al. 1999). In eusocial insects, alterations to the chemical profile may include metabolic by‐products of infection or evolved pheromonal signals that promote altruistic behaviours such as the removal of infected nestmates, resulting in a collective behavioural response known as social immunity (Cremer et al. 2007, 2018). For example, naive leaf‐cutting ants, *Acromyrmex echinatior*, can detect fungal infection in individuals before they become infectious (Walker and Hughes 2009). Infection‐associated chemical cues have thus the potential to influence disease dynamics.

### 1.1 Honey Bee Health and Pathogens

Pollinators, such as honey bees, are vital to global agriculture, providing pollination services to a wide range of plants (Klein et al. 2007; Ollerton 2017; Reilly et al. 2024). They are intensively farmed, eusocial, and heavily reliant on chemical communication (Bortolotti and Costa 2014). They also experience high colony mortality due to pathogens (Potts et al. 2010; Goulson et al. 2015), including bacterial infections such as American foulbrood ( *Paenibacillus larvae* ) (Genersch 2010) and European foulbrood ( *Melissococcus plutonius* ) (Forsgren 2010), Varroa mite ( *Varroa destructor* ) (Traynor et al. 2020), numerous viruses (Gisder and Genersch 2017), and fungal diseases such as chalkbrood (*Ascosphaera apis*) (Aronstein and Murray 2010), and *Vairimorpha* (formerly *Nosema*) spp. (Pasca et al. 2019).

Many honey bee infections are often associated with changes in non‐volatile cuticular hydrocarbons (CHCs), as seen with *Vairimorpha* spp. (McDonnell et al. 2013; Murray et al. 2015), European foulbrood (Kathe et al. 2021), *Varroa destructor* (Wagoner et al. 2019), and Israeli acute paralysis virus (Geffre et al. 2020). Similarly, distinctive VOCs have been found previously to be associated with brood‐stage infections, including American foulbrood (Gochnauer and Margetts 1981; Gochnauer and Shearer 1981; Lee et al. 2020; Bikaun et al. 2022), Varroa mite (Nazzi et al. 2002, 2004; Schöning et al. 2012; Bikaun et al. 2022; Zhao et al. 2025), Chalkbrood (Swanson et al. 2009; Finstrom et al. 2023), and sacbrood virus (Bikaun et al. 2022). However, no studies have investigated VOCs associated with infections in adult bees. This is a critical gap, as adult bees are central to disease transmission dynamics through their roles in nest maintenance and foraging (Fefferman et al. 2007).

### 1.2 *Vairimorpha* Infection in Honey Bees

*Vairimorpha* spp. are microsporidian parasites that infect adult bees, causing Nosemosis disease. Infection occurs following ingestion of spores, which germinate in the midgut and invade epithelial cells, leading to a chronic gut‐associated infection and high spore production that facilitates oral transmission within the colony (Higes et al. 2008). Two species infect honey bees, *Vairimorpha ceranae* and *Vairimorpha apis*, both implicated in colony weakening and collapse (Martín‐Hernández et al. 2018), with *V. ceranae* the dominant species in Europe (Higes et al. 2010). As a gut‐associated infection, *Vairimorpha* spp. can alter host metabolism and are associated with consistent, though modest, shifts in the gut microbiota, including associations with increased abundance of key taxa such as *Gilliamella* (Huang and Evans 2020; Lau et al. 2024; Rubanov et al. 2019; Schwarz et al. 2016). Such changes may influence the host chemical phenotype. Whilst distinctive CHC profiles have been documented in *Vairimorpha*‐infected bees (McDonnell et al. 2013; Murray et al. 2015), VOC alterations remain untested.

Identifying VOCs associated with *Vairimorpha* spp. infection could suggest mechanisms as to how bees detect and respond to infected nestmates but in an applied manner, would support the development of non‐invasive diagnostic tools (Asiri et al. 2024). Effective, scalable diagnostics are needed because beekeepers often lack rapid diagnoses (e.g., PCR) and *Vairimorpha* spp. infections can lack clear clinical symptoms (Holt and Grozinger 2016). Current microscopy‐based field methods are labour‐intensive, unreliable, and disruptive (Botías et al. 2012; Mulholland et al. 2012). If VOC changes precede visible symptoms or spore development, as suggested in leaf‐cutting ants (*A. echinatior*) (Walker and Hughes 2009), they could allow earlier and more effective detection and treatment.

Here, we tested whether *Vairimorpha* spp. infection alters the VOC profile of adult honey bees. Using dynamic headspace sampling over a 14‐day time series, we tracked VOC emissions from infected and uninfected honey bees to determine whether, and when, *Vairimorpha* spp. infection produces a detectable VOC signature.

## 2 Methods

### 2.1 Honey Bee Maintenance

Honey bee ( *Apis mellifera* ) colonies were maintained at the Cardiff University Research Apiary. Prior to collection, all source colonies were screened and confirmed to be free of *Vairimorpha* spp., American foulbrood, and European foulbrood. All colonies exhibited natural levels of *Varroa destructor* infestation at typical UK field levels.

Adult workers of mixed ages were collected directly from super frames in three unrelated colonies and housed in hoarding cages under controlled environmental conditions (33°C, ~65% relative humidity). Each cage consisted of a ventilated plastic deli cup, provisioned *ad libitum* with 50% (w/v) sucrose solution supplied via a modified Eppendorf tube, filter paper to absorb waste, and a sealed VOC sampling port.

### 2.2 *Vairimorpha* spp. Infections

To generate infectious material, adult forager bees were collected from colonies at Fonmon Apiaries (Cardiff, UK) with suspected *Vairimorpha* spp. infection. Infections were confirmed by phase‐contrast microscopy and quantified using a haemocytometer, following standard protocols (Fries et al. 2013).

To prepare the spore suspension, the entire alimentary tract, including midgut, ileum, and rectum, was dissected from infected bees and pooled. Tissues were homogenised in sterile distilled water, and spore purification followed the method described by Fries et al. (2013). Briefly, the homogenate was first filtered through a 70 μm mesh to remove coarse tissue debris. The filtrate was centrifuged (5000× *g* for 5 min) three times, with the pellet re‐suspended in sterile water after each spin. The final spore pellet was re‐suspended in 50% (w/v) sucrose solution to create the infectious inoculum.

Ten hoarding cages (35 bees per cage) were prepared in total. All bees were starved for 1 h prior to feeding to encourage inoculum uptake. Bees in five treatment cages were bulk‐fed with 50% sucrose solution containing 1.75 × 10^7^ *Vairimorpha* spp. spores. This dosage equated to approximately 5 × 10^5^ spores per bee, sufficient to establish high infection. Five control cages received sterile 50% (w/v) sucrose solution. Following inoculation, treatment and control cages were maintained in separate incubators in darkness at 33°C and ~65% relative humidity.

### 2.3 Volatile Collection Across the Infection Time Course

Headspace volatiles (the air volume around bees in a sample bag) were sampled from 5 infected and 5 control hoarding cages at six time points post‐infection: 5 h (day 0), and 3, 6, 9, 12 and 14 days post‐infection (dpi), yielding a total of 30 cage‐level samples per treatment, with one control cage replicate per time point lost due to contamination, and two infected cage replicates at day 6 post‐infection following VOC data processing, resulting in a total of 24 cage replicates for controls and 28 for infected. Each cage was enclosed within a sampling bag constructed from open‐top nalophene bags and sealed. Bags were fitted with a sampling port consisting of a modified Eppendorf tube that connected directly to the interior of the hoarding cage (Figure S1). Bees were left undisturbed in the sealed bag for 30 min to allow VOCs to accumulate within the headspace. A thermal desorption (TD) tube (C2‐EAXX‐5314; inert‐coated SafeLok stainless steel, Markes International) was then inserted into the sampling port and connected to an ACTI‐VOC pump (Markes international) to collect 3 L of air at a flow rate of 200 mL/min (15 min). TD tubes were sealed immediately after collection and stored at ambient temperature until analysis.

At each sample time point, a blank was also collected using an empty hoarding cage enclosed within a sampling bag, containing filter paper and a 50% (w/v) sucrose feeder to control for background emissions.

### 2.4 Infection Monitoring and Validation

Monitoring infection load over time required destructive sampling of bees; therefore, three additional cages of 35 bees (one per colony of origin) were infected as described above in parallel with the VOC‐sampled bees (termed ‘reference’ bees).

Three reference bees were randomly sampled from each cage alongside VOC sampling (0, 3, 6, 9, 12, and 14 dpi), pooled together and *Vairimorpha* spp. spore loads quantified microscopically as described above. All surviving infected bees used in VOC sampling were pooled within cages after the final sampling point (14 dpi). Their average spore loads were measured (*n* = 24 ± 3 bees per cage) and compared with reference bees to confirm infection levels across replicates. All surviving control bees (*n* = 29 ± 3 per cage) were likewise pooled and screened at 14 dpi to ensure no infection had established.

### 2.5 Volatile Sample Analysis

VOC samples were analysed using a thermal desorption two‐dimensional gas chromatography time‐of‐flight mass spectrometry system (TD‐GC × GC–MS), comprising a CENTRI160 inlet system (SepSolve Analytical), an Agilent 8890 GC equipped with a flow modulator (INSIGHT, SepSolve Analytical), and a BenchTOF2‐TI mass spectrometer (Markes International).

Thermal desorption was performed with the CENTRI inlet using the following settings: initial 2 min dry purge with nitrogen at 50 mL/min, followed by two‐stage desorption at 120°C and 280°C for 5 min each, with nitrogen flows of 40 and 50 mL/min, respectively. VOCs were recollected on a trap at 25°C, then desorbed at 300°C for 3 min following a 1 min dry purge with nitrogen (50 mL/min). Total flow during desorption was 2.5 mL/min helium, with 0.5 mL/min directed to the GC and 2.0 mL/min to re‐collection (split ratio 4:1).

Samples were separated on a 20 m × 180 μm, 0.18 μm BPX5 (SGE) as primary column, and a 5 m × 250 μm, 0.1 μm BPX50 (SGE) as secondary column, with 2 s modulation. Carrier gas flow rates (helium) were 0.5 mL/min (1st dimension) and 20 mL/min (2nd dimension) reps. and the temperature programme 40°C for 2 min, 3°C/min to 240°C and 5 min hold at 240°C.

Mass spectra were acquired from m/z 35 to 600 in EI mode at 70 eV at a data rate of 50 Hz. Ion source and transfer line temperature were 230°C and 240°C, respectively.

An injection of 1 μL of a C8 to C20 n‐alkane standard (40 mg/L per component; Supelco) onto a TD tube was analysed under the same conditions and used for Retention index (RI) calculation. RI standards were run before and after (using the re‐collected standard) each set of samples.

### 2.6 Data Processing

Raw chromatograms were processed using *ChromCompare*+ v2.2 (Markes International). All chromatograms were first aligned to the sample containing the highest number of detected peaks followed by dynamic baseline correction with a peak width of 2 s.

A RI pattern was created for subsequent compound integration from a representative chromatogram of the RI standards.

A custom retention‐indexed mass spectral library was created by searching selected chromatograms against the NIST library (2020), using an RI window of ±10 and a medium RI penalty. Compounds that matched both spectra and RI were added as named compounds. Matches with spectral similarity but mismatched RI were classified by chemical class (e.g., “alkane01”), and recurrent, unidentifiable components with consistent RI but no plausible spectral match were categorised as “unknown”.

Peak integration was carried out using the deconvolution function in *ChromCompare*+, with a minimum ion count of 2000, peak merge function, and minimum area of 10,000. Compound identification was performed against the custom‐built, retention‐indexed mass spectral library, with a minimum match factor of 700 for both forward and reverse searches and an RI match window of ±3 RI with a strong RI penalty.

Duplicate entries in integrated peak lists (based on RI) were identified and validated against the original chromatographic data. Where peak splitting by the integrator occurred, duplicate compound names were retained. However, genuinely distinct components (i.e., with similar RI but clearly different spectra) were either renamed to match an existing compound with a similar profile or added to the dataset as new components.

A final compound matrix was constructed containing integrated peak areas for 71 unique compounds. Compounds were retained only if they were detected in at least two of the five replicates within each treatment × timepoint group. This filtering step resulted in two infected replicates at 6 dpi (cages A1 and B2) containing no remaining compounds; these were therefore excluded, leaving three replicates for analysis. Empty cells were imputed with one‐tenth of the smallest non‐zero area in the dataset, and peak areas were normalised within each sample to total peak area.

### 2.7 Statistical Methods

Multivariate analyses of VOC profiles were conducted in R v4.3.2 (R Core Team 2023). All plots were made using *ggplot2* (Wickham 2011). Normalised VOC abundances were used as the response variable throughout.

Permutational multivariate analysis of variance (PerMANOVA) with 999 permutations and Euclidean distances (adonis2 function in ‘vegan’, Oksanen et al. 2025) was used to test differences in VOC profiles between treatment (control vs. infected) and between treatment, dpi, and their interaction.

Canonical analysis of principal coordinates (CAP, CAPdiscrim function in *BiodiversityR*, Kindt and Coe 2005) and Random forest (RF, package *randomForest*, Liaw and Wiener 2002) were used to evaluate the discriminatory power of VOC profiles between treatment and dpi combinations. We used CAP models with Euclidean distances and RF to assess: (i) treatment (control vs. infected) as a variable, to assess overall differences pooled across all time points; and (ii) a combined factor of treatment and day post‐infection (dpi), to assess how infected and control VOC profiles diverged over time.

The mean decrease accuracy in RF was used to identify compounds most important for classifying by defining a threshold based on the inflection point in the ranked top importance distribution, corresponding to the transition between strongly and weakly informative VOCs (Figure S2). This approach follows established random forest variable selection methods that identify informative predictors from ranked importance values (Genuer et al. 2010). The top ranked VOCs were re‐run in CAP to test whether the subset improved separation.

As a complementary approach for identifying compounds most important for classifying, redundancy analysis (RDA, ‘vegan’ package Oksanen et al. 2025) was performed with treatment, dpi, and their interaction as predictor variables. Magnitude, as the Euclidean distance of each compound vector from the origin, of individual VOCs, was used to rank components (Legendre and Legendre 2012). A threshold was defined at the inflection point in the ranked distribution of magnitudes, as done for the random forest analysis. This approach is analogous to identifying points of diminishing returns in ranked multivariate data (Cattell 1966; Jackson 1993; Figure S3).

VOCs consistently ranked as important across both approaches were considered key compounds and the most robust signals of infection. We assessed the significance of overall model fit, individual predictors, and constrained axes using permutation tests (‘anova.cca’ in *vegan*, 999 permutations). To compare across key VOCs with different baseline levels, we computed *Z*‐scores.

## 3 Results

### 3.1 *Vairimorpha* spp. Infections

*Vairimorpha* spp. infections progressed rapidly across all replicates. Reference bees sampled destructively at 0–14 dpi showed typical infection trajectories, as reported previously (Forsgren and Fries 2010; Huang and Solter 2013; Fan et al. 2024). Spore loads rose from 6.0 ± 2.8 × 10^4^ at 3 dpi to 2.0 ± 0.27 × 10^6^ at 6 dpi reaching 2.7 ± 0.18 × 10^7^ at 9 dpi, and exceeding 10^8^ spores per bee by 12 dpi. At 14 dpi, both reference and experimental bees exhibited similarly high spore loads (1.18 ± 0.14 × 10^8^), confirming a comparable infection trajectory (Figure S4). All but one of the controls were free of *Vairimorpha* spores at 14 dpi. The contaminated control was excluded from all analyses.

### 3.2 Mortality

Mortality was broadly similar between treatments across the 14‐day experiment (Figure S5). The mean number of bees alive per cage declined over time in both infected and uninfected bees, with no consistent divergence in survival trajectories. Variation among cages was high but comparable between treatments at all time points (Figure S5).

### 3.3 Separation of Infected and Control VOC Profiles Pooled Across Time

A total of 71 VOCs were found across both infected and uninfected treatments over the 14‐day time series (Table S1). No VOCs were specific to either control or infected bees, but the relative abundance and pattern of VOCs significantly differed between control and infected bees when pooled across all time points (PerMANOVA: *p* = 0.02, *R* ^2^ = 0.038). At 6 dpi, only three infected replicates remained after filtering, as none of the VOCs detected in two replicates (cages A1 and B2) were present in at least two of the five replicates and were therefore removed.

**FIGURE 1:**
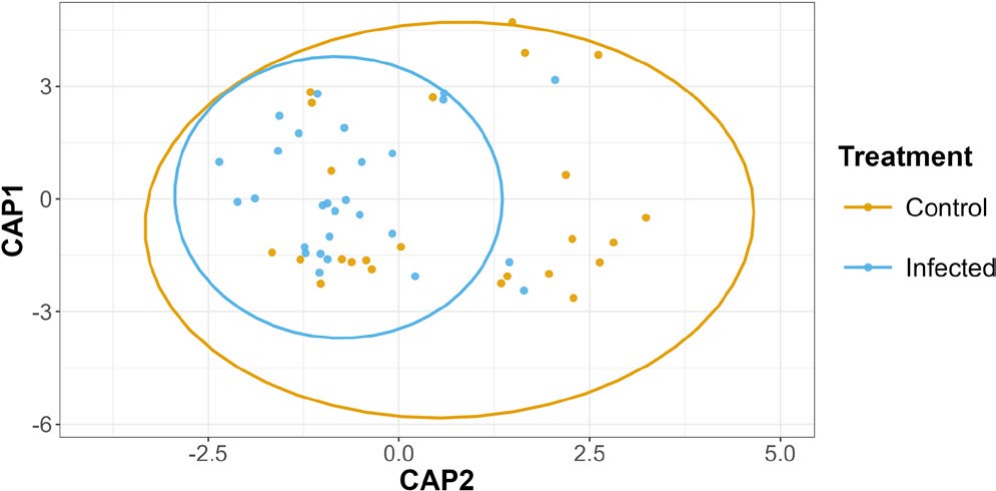
Samples projected onto the first two axes of a canonical analysis of principal coordinates (CAP) based on profiles of all 71 volatile organic compounds (VOCs), comparing treatments across all days post‐infection (DPI). Points represent individual samples coloured by treatment. Ellipses denote 95% confidence intervals around group centroids. Each point represents an individual cage replicate (control *n* = 24, infected *n* = 28).

Infected bees were correctly classified 85.7% of the time, compared with only 50% for controls. Infected bees clustered more tightly, and control samples were more dispersed, leading to a considerable overlap with the infected group (Figure 1).

### 3.4 Temporal Changes in VOC Profiles During Infection

VOC profiles were significantly different between treatments (PerMANOVA: *p* = 0.002, *R* ^2^ = 0.038), dpi (PerMANOVA: *p* = 0.001, *R* ^2^ = 0.31), and their interaction (PerMANOVA: *p* = 0.003, *R* ^2^ = 0.11). Over time, differences were strongest at 12 dpi (CAP: 100% for controls, 80% for infected), moderate at 6–9 dpi (CAP: 33%–60%), and at 0 and 14 dpi infected and control bees were indistinguishable (CAP: 0%; Table S2).

**FIGURE 2:**
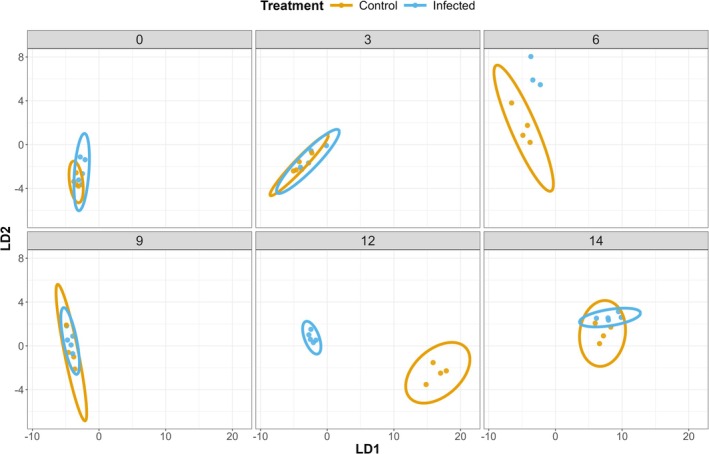
Linear discriminant plot from canonical analysis of principal coordinates of the 12 most important volatile organic compounds (VOCs) from random forests in adult worker honey bees of mixed ages, infected with *Vairimorpha* spp. or uninfected, across days post‐infection (dpi). Ellipses represent 95% confidence intervals. Confidence intervals could not be computed at 6 dpi as only 3 replicates were retained after VOC data processing. Each point represents a cage replicate. At 6 dpi control (*n* = 4); infected (*n* = 3). At all other time points control (*n* = 4), infected (*n* = 5).

Random forest identified a subset of 12 VOCs that most strongly affected discrimination between infected and uninfected bees across time (Figure S2). This reduced set of VOCs explained more of the overall variance (72% compared to 46% with all 71 VOCs) in PerMANOVA and improved CAP classification accuracy by 21% (Figure 2; Table S3). Using this identified VOC subset, infected and control bee VOC profiles were indistinguishable at 0–3 dpi, distinct at 6 dpi (CAP: 100% accuracy for infected, 75% control), converged at 9 dpi, diverged at 12 dpi (CAP: 100% accuracy for both; Table S3), and converged at 14 dpi (Figure 2), suggesting infection signals emerge only at certain time periods of the infection.

### 3.5 Compound Associations With Infection Stages

**FIGURE 3:**
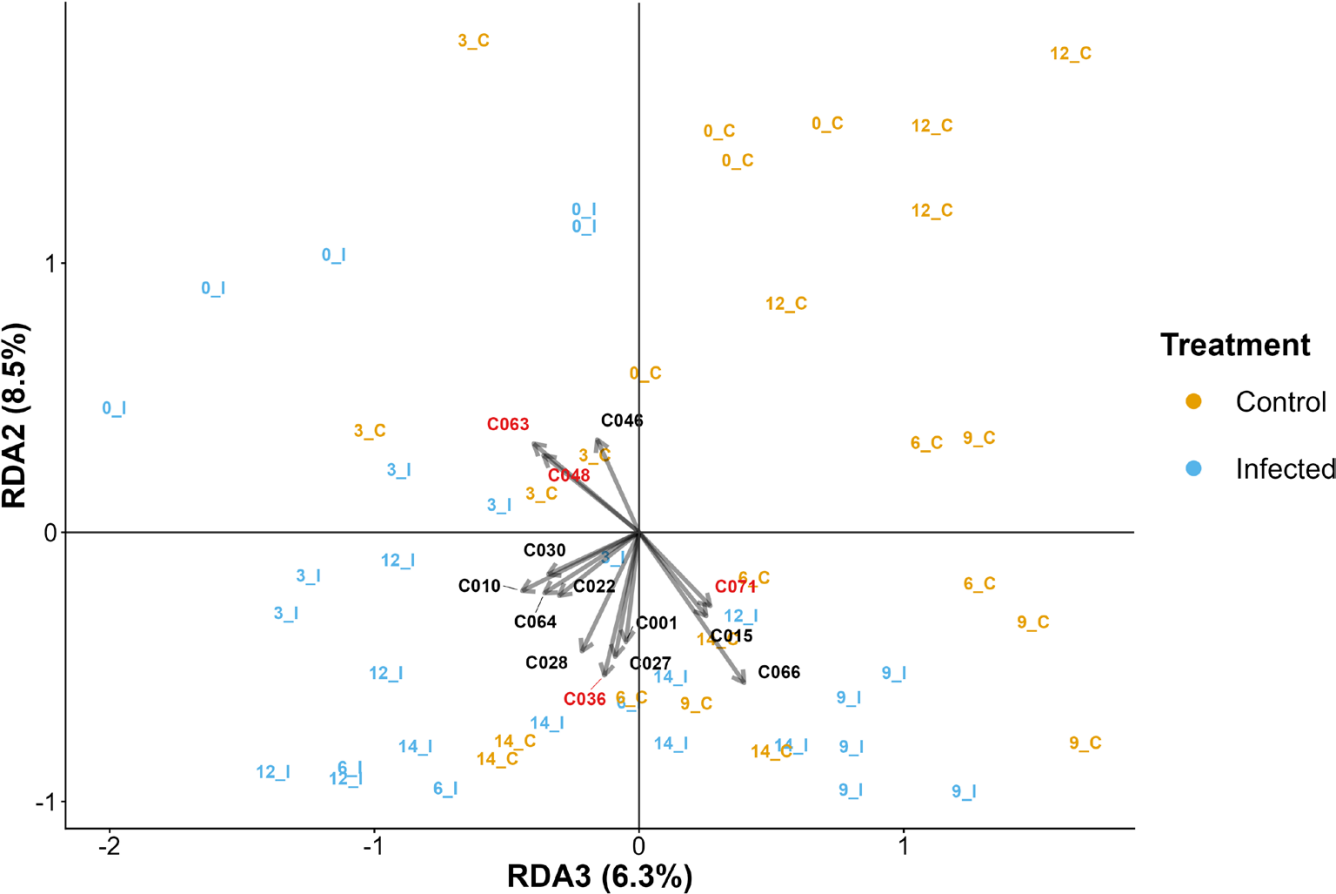
Redundancy analysis (RDA) biplot of VOC profiles, of 71 identified volatile organic compounds (VOCs) constrained by treatment, days post‐infection (dpi), and their interaction. Sample IDs indicate an individual sample taken at a combined dpi and treatment (e.g., 3_I = dpi 3, infected treatment) for both control (*n* = 24 cage replicates) and infected (*n* = 28 cage replicates). The top VOCs contributing to constrained variance show on the plot with arrows indicating how strongly a compound is correlated with the constrained axes. VOCs not strongly contributing to constrained variance are not shown. Top compounds with consensus between random forest and RDA are shown in red.

Redundancy analysis (RDA) confirmed that treatment, days post‐infection (dpi), and their interaction significantly shaped VOC profiles (treatment: *F*_1,40_ = 2.8, *p* = 0.001; dpi: *F*_5,40_ = 4.6, *p* = 0.001; treatment*dpi: *F*_5,40_ = 1.6, *p* = 0.004) and explained 31% of the total variance in VOC profiles (adjusted *R* ^2^). The first four RDA axes (RDA1‐RDA4) were significant (*p <* 0.02), and accounted for 16.5%, 8.5%, 6.4%, and 5.1% of the total unadjusted variance, respectively. Sample scores were strongly ordered by dpi along RDA1, RDA2 primarily separated infected and control bees, and RDA3 reflected their interaction. We plotted RDA2 against RDA3 to highlight separation driven by treatment and the variation over time (Figure 3).

Infected bees at 0 and 3 dpi clustered in the quadrant of positive RDA2 and negative RDA3, overlapping with 0 dpi controls (Figure 3) and were linked with tetradecane (C063), dodecane (C048), and decane (C046). The positive RDA3 and negative RDA2 quadrant primarily contained both mid‐late stage (6–12 dpi) infected and control bees and was associated with undecane (C066), Alkane07 (C015), and an unidentified compound (C071) (Figure 3). The quadrant of both negative axes was almost exclusively occupied by infected samples from multiple stages of infection (3–14 dpi), apart from two controls at 14 dpi that overlapped with infected bees. This region was characterised by benzaldehyde, 3,4‐dimethyl‐ (C036), 3‐ethyl‐3‐methylheptane (C010), 1‐octanol, 2,2‐dimethyl‐ (C001), several long‐chain alkanes (C030, C028, C027, C022), and toluene (C064).

### 3.6 Identifying Robust Markers of Infection

**FIGURE 4:**
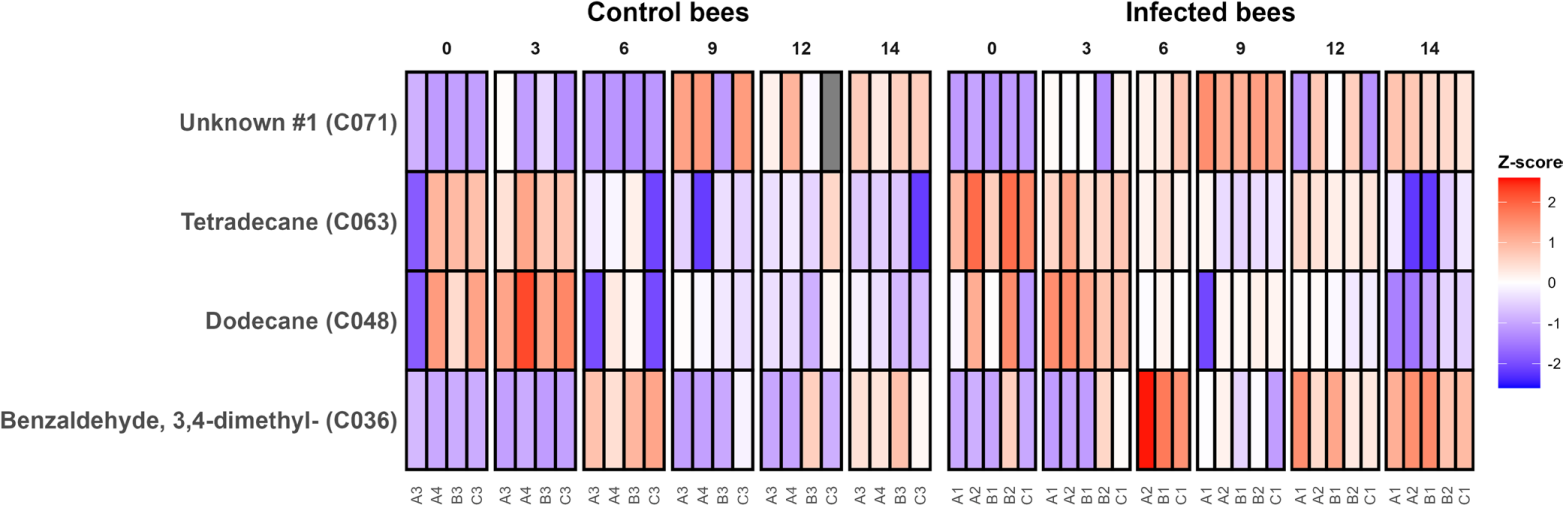
Heatmap of standardised abundances of 4 consensus (RF and RDA) volatile organic compounds in adult honey bee workers across a 14‐day post‐infection time series. Each tile represents the *Z*‐scored abundance of a compound (row) in each sample (column) at a given day post‐infection (dpi). Panels are faceted by treatment group (control vs. *Vairimorpha* spp.‐infected bees), sample labels indicate original donor hive (A, B, or C) and cage ID. *Z*‐scores were calculated separately for each compound across all samples to indicate relative increases (red) and decreases (blue) in abundance.

A consensus between RDA and random forest identified four compounds consistently associated with treatment separation: tetradecane (C063), dodecane (C048), benzaldehyde, 3,4‐dimethyl‐ (C036), and one unknown compound (C071) (Figure 4). Across both treatments, tetradecane and dodecane showed similar temporal patterns, with relatively higher abundances at early time points (0–3 dpi) followed by a steady decline towards 14 dpi. This decline was evident in both control and infected bees, although infected bees tended to show higher tetradecane at 0 dpi and lower dodecane at 3 dpi. In contrast, benzaldehyde, 3,4‐dimethyl‐ showed a distinct increase over time in infected bees, rising markedly by 6 dpi and remaining elevated at later time points. This increase was less pronounced in control bees, resulting in a divergence between treatments over the course of infection. The unknown compound (C071) also exhibited treatment‐specific temporal dynamics, increasing from 3 dpi onwards in infected bees, whereas in control bees increases were delayed until 9 dpi, after which abundances converged between treatments. Together, these patterns indicate that treatment differences were not restricted to single time points but reflected differences in the trajectories of VOC abundance across the infection time series.

## 4 Discussion

We show for the first time that infection of adult honey bees by *Vairimorpha* spp. alters the VOC profile in a detectable way. All VOCs identified were emitted by both control and infected bees, but their relative abundances and patterns of emission varied between treatments. VOC profiles differed strongly between infected and control bees at 6‐ and 12‐days post‐infection. Four compounds—tetradecane, dodecane, 3,4‐dimethylbenzaldehyde, and one unidentified compound—consistently discriminated between infected and uninfected bees. Our findings provide a foundation for understanding how volatile cues vary during *Vairimorpha* spp. infection and highlight potential biomarkers of infection.

### 4.1 Variation in VOC Emissions Overall and Across Time

VOC profiles of infected and uninfected bees significantly differed when pooled across time points, in line with previous studies reporting changes in low volatility CHCs during *Vairimorpha* infection (Murray et al. 2015; McDonnell et al. 2013). We found that infection‐associated VOC changes were not linear but instead discrimination peaked at 6 and 12 dpi, with convergence at 9 and 14 dpi. Interestingly, McDonnell et al. (2013) also reported a non‐linear relation of CHC profiles between *Vairimorpha*‐infected and uninfected bees but at different time points to our observations. CHC profiles converged at 5 dpi and became distinct at 10 dpi. Our results suggest VOCs may act as earlier, colony‐level cues of infection, whereas CHCs provide later, contact‐based confirmation, together forming complementary channels of chemical communication. As CHCs are non‐volatile, they require direct contact and typically convey identity‐related information such as nestmate recognition or division of labour (Drijfhout et al. 2009). In contrast, VOCs are volatile, act over longer distances, and can serve both as communication signals and as cues tied to host or pathogen metabolism, meaning they could elicit different responses in nestmates than CHCs.

Because VOCs disperse through the hive atmosphere, they may not only mediate interactions with specific individuals but also provide colony‐wide information on infection risk. Rather than triggering targeted behaviours such as removal, these cues could influence broader prophylactic or defensive responses across the colony. For example, exposure to infection‐associated volatiles has the potential to prime immune responses in naïve individuals or to alter collective behaviours that reduce pathogen transmission risk. Colony‐level responses such as increased propolis use or changes in defensiveness have been shown to occur in response to pathogen pressure (Simone‐Finstrom and Spivak 2012; Simone‐Finstrom et al. 2017), and may be mediated in part by volatile cues. One possible explanation for the difference in VOC profiles between treatments as time progressed is that infection altered the pace of normal age‐related changes in odour profile, analogous to precocious behavioural physiological changes induced by *Vairimorpha* infection (Goblirsch et al. 2013). However, our study used workers of mixed ages collected from super frames, meaning we cannot make predictions on how the age of the bees might have influenced VOC emissions over time. Although, as all replicates consisted of equally random assortments of ages, it is more likely that the treatments influenced VOC profiles over time.

Our findings of divergence and convergence in VOC profiles over time may be explained by *V. ceranae* infection dynamics. Previous studies have reported an infection trajectory with rapid increases in spore load between 4 and 8 dpi, followed by a temporary lag phase around 8 and 10 dpi, and a subsequent increase between 10 and 14 dpi, where spore proliferation levels off between 14 and 18 dpi (Forsgren and Fries 2010; Huang and Solter 2013; Fan et al. 2024). Our sampling interval (every 3 days) was insufficient to resolve the lag phase between 8 and 10 dpi explicitly in spore counts. However, our overall infection trajectory was consistent with previous findings, and this framework may help explain the temporal pattern in VOC profiles. The convergence in VOC profiles at 9 and 14 dpi occurred at the point when other studies suggest parasite proliferation temporarily slows, whereas the divergence observed at 6 and 12 dpi coincided with periods of rapid parasite replication (Forsgren and Fries 2010; Huang and Solter 2013; Fan et al. 2024). Because spore proliferation is minimal in the first few days post‐infection, the absence of VOC divergence at 0–3 dpi is likewise consistent with low parasite abundance. Together, these patterns suggest that volatile cues may be most strongly expressed during periods of rapid parasite replication, and reduced during transitional phases when replication temporarily slows. Previous transcriptomic analyses of *Vairimorpha* infecting honey bees support this hypothesis, showing they differentially express metabolic genes at distinct time points during infection (Badaoui et al. 2017; Fan et al. 2022; Li et al. 2022).

By 14 dpi, VOC profiles had become indiscriminate. While this may reflect ongoing metabolic changes in the host–parasite interaction, it is also plausible that prolonged caging induced stress in the uninfected bees, which can alter physiology and metabolism, and could have affected VOC emissions (Alburaki et al. 2019; Lattorff 2022). Mortality rates were comparable between treatments throughout the experiment (Figure S5), suggesting that the effects of caging stress were similar between infected and uninfected bees, and are unlikely to explain earlier divergence in VOC profiles, but it could explain the convergence at 14 dpi. Although specific VOC signatures of caging stress have not been characterised, prolonged confinement could induce metabolic changes that influence volatile emissions in the uninfected bees. If caging and infection impose similar physiological stress, they may drive convergent changes in VOC profiles, reducing the distinction between infected and uninfected bees at later time points. To further resolve the temporal dynamics underlying divergence and convergence in VOC profiles, future work should incorporate higher‐resolution sampling across the infection period, including daily timepoints beyond 14 dpi. Quantifying spore loads directly after VOC sampling at each timepoint would allow direct linking of parasite load, volatile emissions, and infection dynamics. Nevertheless, the link between VOC profile differences and *Vairimorpha* growth dynamics in the mid‐stage infections indicates that VOC profiles reflect dynamic host–parasite metabolic interactions, rather than static infection status.

### 4.2 Robust Markers and Shifts in VOC Profiles

Four compounds were identified with both Random Forest and RDA as drivers of separation of VOC profiles of infected from uninfected bees. Tetradecane and dodecane are common components of honey bee brood VOC profiles (Light et al. 2020; Liendo et al. 2021; Liu et al. 2022). 3,4‐dimethylbenzaldehyde has not been reported from honey bees, but benzaldehyde itself occurs in brood volatiles (Schöning et al. 2012; Liolios et al. 2022), and related derivatives possess antimicrobial properties (Kim et al. 2019).

The relatively higher abundance of tetradecane in infected bees at 0 dpi appeared to drive separation at this time point and suggests it may be emitted at elevated levels immediately following infection. Tetradecane strongly triggers hygienic behaviour in *Varroa*‐infested brood (Noël et al. 2025), making its early association with infection a plausible cue for removal by the host colony before spores mature and become infectious. However, its functional role in adults remains untested. *Vairimorpha* spores can germinate within 30 min of ingestion (Goblirsch 2018), suggesting that either host or parasite metabolic changes could initiate this early cue production.

Dodecane followed the same general abundance and clustering patterns as tetradecane. It is a common honey bee alkane, though it does not trigger aggression when tested in isolation (Breed and Stiller 1992). The reduction of tetradecane and dodecane as infection progresses could indicate that these VOCs may play a role in triggering removal prior to spore maturation, or alternatively a co‐evolutionary adaptation where the infection itself suppresses host signalling capacity. Indeed, *V. ceranae* is known to suppress host immune responses and down‐regulate cuticle genes and odorant binding proteins (Badaoui et al. 2017).

While we did not test behavioural responses directly, the differences in VOCs between treatments demonstrate that infection alters the odour landscape of bees. Whether these VOCs function as signals, cues, or metabolic by‐products remains unclear, particularly as they may originate from the host, associated microbiota, or the parasite itself. Alternative explanations should therefore be considered, including the possibility that these compounds arise from infection‐induced physiological damage or represent parasite‐mediated changes that enhance transmission rather than host defence. This has been seen in other honey bee infections; for example, Israeli acute paralysis virus alters host chemical cues in such a way that inter‐colonial transmission becomes more likely (Geffre et al. 2020). Manipulating host scent could also lead to increased grooming or other behavioural responses that facilitate spore dispersal. Increased grooming in response to *Vairimorpha* infection has been seen previously (Biganski et al. 2017), and this could be triggered by shifts in VOCs, but whether this leads to downstream reduction or increase in infection is unknown. Alterations in VOC profile may also reflect gut microbiota disruption associated with infection (Huang and Evans 2020; Lau et al. 2024; Rubanov et al. 2019; Schwarz et al. 2016). Furthermore, as *V*. *ceranae* is thought to represent a relatively recent host shift from Asian honey bees (*Apis cerana*) (Paxton et al. 2007), tightly coevolved signalling mechanisms may not be established.

Nonetheless, such changes could, in principle, influence colony‐level processes. Evidence from other systems supports this hypothesis. In mice, exposure to odours from diseased conspecifics reduces innate immune responsiveness in uninfected individuals (Alves et al. 2010). In insects (*Drosophila* spp.), exposure to parasitoid wasp odours primes immune responses against future challenges (Madhwal et al. 2020). Whether infection‐associated VOCs in adult honey bees induce comparable behavioural or immune responses remains unknown. Within this context, the higher association of 3,4‐dimethylbenzaldehyde with later‐stage infections in our study, alongside reductions in tetradecane, may reflect a shift in host physiology as infection progresses. Although 3,4‐dimethylbenzaldehyde has not previously been identified in honey bees, benzaldehyde is present in brood, propolis and wax (Schöning et al. 2012; Liolios et al. 2022; Smith and Bromenshenk 2002), and related derivatives have antimicrobial properties (Kim et al. 2019), suggesting that it could represent a host‐derived compound or a gut microbiota by‐product associated with late‐stage infection. Because social immunity can be costly, one possibility is that these changes reflect a reallocation of resources from colony‐level defences to individual‐level responses as infection intensifies (Cremer et al. 2007; Cotter and Kilner 2010a, 2010b). However, establishing the functional role and biological origin of these compounds will require direct behavioural assays and mechanistic studies.

Although our methods were robust for distinguishing between different VOCs, their identities were not verified against reference standards, and functional interpretations should therefore be made with caution. Nevertheless, absolute identification of diagnostic markers may not be necessary. Bees themselves may detect infection as a deviation from the normal colony odour template (Tibbetts and Dale 2007; Gherardi et al. 2012), and gas‐sensor technologies could likewise be trained to recognise whole‐profile variation rather than specific compounds (Asiri et al. 2024).

### 4.3 Can VOCs be Used for Disease Surveillance?

Transient shifts in VOC profiles could mean that infection is only detectable at specific stages, such as 6 or 12 dpi. However, colony infections are rarely synchronous. Individual bees differ widely in spore load and stage of infection (Mulholland et al. 2012), meaning that colony‐level headspace reflects a composite of multiple infection stages. In this context, analysing whole‐colony odour and differentiating it from that of a healthy colony is likely to provide a more consistent and reliable signal than attempting to track stage‐specific markers, and could therefore offer a practical estimation of infection at the colony level. In practice, colony headspace is chemically complex, containing not only bee‐derived volatiles but also odours from hive products such as honey and pollen, as well as VOCs from hive materials including wood and paint (Smith and Bromenshenk 2002). Any infection signal must therefore be strong enough to stand out against this background noise. This makes identifying bouquets of VOCs that consistently contribute to treatment discrimination especially valuable. While unique biomarkers would be valuable in principle (Bikaun et al. 2022), they may not always exist if infection alters compounds already present in the baseline odour rather than producing unique metabolites (Asiri et al. 2024). Profile‐level shifts are therefore particularly important because they can still be exploited even when specific biomarkers are absent. In our study, no VOCs were found to be uniquely emitted by *Vairimorpha*‐infected bees compared with uninfected bees, but relative abundances and overall patterns changed at a profile level. In such cases, training gas sensors to recognise multivariate patterns of VOCs provides a more realistic and effective approach.

## 5 Conclusions

Our study demonstrates that *Vairimorpha* spp. infection alters the volatile profile of adult honey bees in ways that are both detectable and dynamic. Although variation and overlap were present, distinct differences emerged at specific infection stages, particularly 6‐ and 12‐days post‐infection, coinciding with rapid parasite replication. Rather than a single biomarker, subsets of compounds consistently contributed to treatment discrimination, suggesting that infection odours act as profile‐level signals. These findings have two main implications. First, they support the development of gas sensor technologies for non‐invasive disease surveillance, where recognising whole‐profile shifts may be more realistic than relying on unique compounds. Second, they highlight a potential role for infection odours in regulating social immunity, with temporal VOC shifts potentially shaping when and how nestmates respond to diseased individuals. However, whether adults can detect and respond to the shift in VOC profiles associated with infection found here requires mechanistic behavioural studies. Our results provide a foundation for applied surveillance tools and a framework for testing how chemical cues underpin social immunity—not only in bees but across all social animals.

## Author Contributions

**Ayman Asiri:** conceptualization (lead), data curation (lead), formal analysis (lead), funding acquisition (equal), investigation (lead), methodology (lead), project administration (equal), visualization (lead), writing – original draft (lead), writing – review and editing (lead). **Sarah E. Perkins:** conceptualization (equal), data curation (supporting), formal analysis (supporting), funding acquisition (equal), investigation (equal), methodology (equal), project administration (equal), resources (equal), supervision (equal), writing – review and editing (equal). **Carsten T. Müller:** conceptualization (equal), data curation (equal), formal analysis (supporting), funding acquisition (equal), investigation (equal), methodology (equal), project administration (equal), resources (equal), software (lead), supervision (equal), writing – review and editing (equal).

## Funding

This work was supported by the Natural Environment Research Council (NE/S007504/1) and Markes International through the GW4+ DTP iCASE Studentship (2021), Markes International Ltd., and Bee Diseases Insurance Ltd. (BDI).

## Conflicts of Interest

The authors declare no conflicts of interest.

## Supporting information


**Figure S1:** Custom headspace sampling bags consisting of a hoarding cage, 50% w/v sucrose feeder, and Eppendorf sampling port connected to the interior of the cage. 3 L of headspace was extracted onto thermal desorption tubes using an ACTI‐VOC pump at 200 mL/min.
**Figure S2:** Top 30 volatile organic compounds (VOCs) ranked by their importance to classification accuracy in the random forest model based on all 71 VOCs. Higher values indicate a greater contribution to model performance. The dashed red line indicates the cut‐off point used to select the most important compounds for discriminating between combined treatment and days post‐infection (dpi) variables, defined by the largest step change in mean decrease accuracy.
**Figure S3:** Top 30 volatile organic compounds (VOCs) ranked by their contribution to the redundancy analysis (RDA) model based on all 71 VOCs. Higher magnitudes indicate a stronger contribution to constrained variance. The dashed red line indicates the cut‐off point used to select the most important compounds for discriminating between combined treatment and days post‐infection (dpi) variables.
**Figure S4:** Infection dynamics of *Vairimorpha* spp. in reference and experimental bees. Reference bees were destructively sampled at 0, 3, 6, 9, 12, and 14 days post‐infection (dpi) to confirm infection progression, while experimental bees used for volatile organic compound sampling were only sampled at 14 dpi. Each point represents a pooled sample of three bees. The black line and shaded ribbon show a loess smoother with ±95% confidence interval fitted across all data. Due to natural mortality, only one reference cage replicate was available at 12 and 14 dpi. Spore loads rose from 6.0 ± 2.8 × 10^4^ at 3 dpi to 2.0 ± 0.27 × 10^6^ at 6 dpi reaching 2.7 ± 0.18 × 10^7^ at 9 dpi, and exceeded 10^8^ spores per bee by 12 dpi. At 14 dpi, both reference and experimental bees exhibited similarly high spore loads (1.18 ± 0.14 × 10^8^).
**Figure S5:** Number of bees alive per cage across the 14‐day experiment for control and infected treatments. Thin lines represent individual cages (labels shown at day 14); thick lines show treatment means. Mortality declined over time in both treatments, with similar trajectories despite variation among cages.
**Table S1:** The 71 volatile organic compounds (VOCs) identified in infected and uninfected bees across all timepoints. Retention indices (RI) were calculated from experimental data and compared against literature values from the NIST 2020 mass spectral library. Where multiple database entries were available, the experimental RI value for non‐standard non‐polar columns was adopted for consistency throughout, although column‐specific values were available in some cases.
**Table S2:** Classification success (%) from canonical analysis of principal coordinates (CAP) using all 71 VOCs across treatments and days post‐infection. CAP achieved an overall classification success of 46%.
**Table S3:** Classification success (%) from canonical analysis of principal components (CAP) using the top 12 VOCs identified as most important in the random forest model across treatments and days post‐infection. CAP achieved an overall classification success of 67%.

## Data Availability

Data and code available from Dryad digital repository: https://doi.org/10.5061/dryad.ttdz08mc3.

## References

[ece373768-bib-0001] Ahmed, W. M. , O. Lawal , T. M. Nijsen , R. Goodacre , and S. J. Fowler . 2017. “Exhaled Volatile Organic Compounds of Infection: A Systematic Review.” ACS Infectious Diseases 3, no. 10: 695–710. 10.1021/acsinfecdis.7b00088. 28870074

[ece373768-bib-0002] Alburaki, M. , S. Karim , K. Lamour , J. Adamczyk , and S. D. Stewart . 2019. “RNA‐Seq Reveals Disruption of Gene Regulation When Honey Bees Are Caged and Deprived of Hive Conditions.” Journal of Experimental Biology 222, no. 18: jeb207761. 31413101 10.1242/jeb.207761 PMC7376871

[ece373768-bib-0003] Alves, G. J. , L. Vismari , R. Lazzarini , J. L. Merusse , and J. Palermo‐Neto . 2010. “Odor Cues From Tumor‐Bearing Mice Induces Neuroimmune Changes.” Behavioural Brain Research 214, no. 2: 357–367. 10.1016/j.bbr.2010.06.003. 20541567

[ece373768-bib-0004] Aronstein, K. A. , and K. D. Murray . 2010. “Chalkbrood Disease in Honey Bees.” Journal of Invertebrate Pathology 103, no. Suppl 1: S20–S29. 10.1016/j.jip.2009.06.018. 19909969

[ece373768-bib-0005] Asiri, A. , S. E. Perkins , and C. T. Müller . 2024. “The Smell of Infection: Disease Surveillance in Insects Using Volatile Organic Compounds.” Agricultural and Forest Entomology 27, no. 1: 81–89. 10.1111/afe.12651.

[ece373768-bib-0006] Badaoui, B. , A. Fougeroux , F. Petit , et al. 2017. “RNA‐Sequence Analysis of Gene Expression From Honeybees ( *Apis mellifera* ) Infected With *Nosema ceranae* .” PLoS One 12, no. 3: e0173438. 10.1371/journal.pone.0173438. 28350872 PMC5370102

[ece373768-bib-0092] Biganski, S. , C. Kurze , M. Y. Müller , and R. F. A. Moritz . 2017. “Social Response of Healthy Honeybees Towards *Nosema ceranae*‐Infected Workers: Care or Kill?” Apidologie 49, no. 3: 325–334. 10.1007/s13592-017-0557-8.

[ece373768-bib-0007] Bikaun, J. M. , T. Bates , M. Bollen , et al. 2022. “Volatile Biomarkers for Non‐Invasive Detection of American Foulbrood, a Threat to Honey Bee Pollination Services.” Science of the Total Environment 845: 157123. 10.1016/j.scitotenv.2022.157123. 35810895

[ece373768-bib-0008] Blomquist, G. J. , C. Tittiger , and R. Jurenka . 2020. “Cuticular Hydrocarbons and Pheromones of Arthropods.” In Hydrocarbons, Oils and Lipids: Diversity, Origin, Chemistry and Fate, 213–244. Springer International Publishing.

[ece373768-bib-0009] Bortolotti, L. , and C. Costa . 2014. “Chemical Communication in the Honey Bee Society.” In Neurobiology of Chemical Communication. CRC Press/Taylor & Francis. 24830041

[ece373768-bib-0010] Botías, C. , R. Martín‐Hernández , A. Meana , and M. Higes . 2012. “Critical Aspects of the *Nosema* spp. Diagnostic Sampling in Honey Bee ( *Apis mellifera* L.) Colonies.” Parasitology Research 110: 2557–2561. 22193523 10.1007/s00436-011-2760-2

[ece373768-bib-0011] Breed, M. D. , and T. M. Stiller . 1992. “Honey Bee, *Apis mellifera* , Nestmate Discrimination: Hydrocarbon Effects and the Evolutionary Implications of Comb Choice.” Animal Behaviour 43, no. 6: 875–883.

[ece373768-bib-0012] Calcagnile, M. , S. M. Tredici , A. Tala , and P. Alifano . 2019. “Bacterial Semiochemicals and Transkingdom Interactions With Insects and Plants.” Insects 10, no. 12: 441. 10.3390/insects10120441. 31817999 PMC6955855

[ece373768-bib-0013] Cattell, R. B. 1966. “The Scree Test for the Number of Factors.” Multivariate Behavioral Research 1, no. 2: 245–276. 10.1207/S15327906MBR0102_10. 26828106

[ece373768-bib-0015] Cotter, S. C. , and R. M. Kilner . 2010b. “Sexual Division of Antibacterial Resource Defence in Breeding Burying Beetles, *Nicrophorus vespilloides* .” Journal of Animal Ecology 79, no. 1: 35–43. 10.1111/j.1365-2656.2009.01593.x. 19627394

[ece373768-bib-0014] Cotter, S. , and R. Kilner . 2010a. “Personal Immunity Versus Social Immunity.” Behavioral Ecology 21, no. 4: 663–668.

[ece373768-bib-0016] Cremer, S. , S. A. Armitage , and P. Schmid‐Hempel . 2007. “Social Immunity.” Current Biology 17, no. 16: R693–R702. 10.1016/j.cub.2007.06.008. 17714663

[ece373768-bib-0017] Cremer, S. , C. D. Pull , and M. A. Furst . 2018. “Social Immunity: Emergence and Evolution of Colony‐Level Disease Protection.” Annual Review of Entomology 63, no. 1: 105–123. 10.1146/annurev-ento-020117-043110. 28945976

[ece373768-bib-0018] Drijfhout, F. P. , R. Kather , and S. J. Martin . 2009. “The Role of Cuticular Hydrocarbons in Insects.” In Behavioral and Chemical Ecology, edited by H. L. Wen Zhang , 91–114. Nova Science Publishers, Inc.

[ece373768-bib-0019] Ehman, K. , and M. Scott . 2001. “Urinary Odour Preferences of MHC Congenic Female Mice, *Mus domesticus* : Implications for Kin Recognition and Detection of Parasitized Males.” Animal Behaviour 62, no. 4: 781–789.

[ece373768-bib-0020] Fan, X. , H. Zhao , H. Zang , et al. 2024. “Extensive Influence of Microsporidian Infection on Sucrose Solution Consumption, Antioxidant Enzyme Activity, Cell Structure, and Lifespan of Asian Honeybees.” Frontiers in Immunology 15: 1404766. 10.3389/fimmu.2024.1404766. 39628478 PMC11611804

[ece373768-bib-0021] Fan, Y. , J. Wang , K. Yu , et al. 2022. “Comparative Transcriptome Investigation of *Nosema ceranae* Infecting Eastern Honey Bee Workers.” Insects 13, no. 3: 241. 10.3390/insects13030241. 35323539 PMC8952433

[ece373768-bib-0022] Fefferman, N. H. , J. F. Traniello , R. B. Rosengaus , and D. V. Calleri . 2007. “Disease Prevention and Resistance in Social Insects: Modeling the Survival Consequences of Immunity, Hygienic Behavior, and Colony Organization.” Behavioral Ecology and Sociobiology 61, no. 4: 565–577.

[ece373768-bib-0023] Finstrom, M. S. , M. Angove , P. Brooks , and J. Gerdts . 2023. “Identification and Discrimination of Volatiles Associated With Chalkbrood Infection in European Honey Bees ( *Apis mellifera* ), Eastern Australia.” Preprint, Research Square Company 24. 10.21203/rs.3.rs-2690582/v1.

[ece373768-bib-0024] Forsgren, E. 2010. “European Foulbrood in Honey Bees.” Journal of Invertebrate Pathology 103, no. Suppl 1: S5–S9. 10.1016/j.jip.2009.06.016. 20105559

[ece373768-bib-0025] Forsgren, E. , and I. Fries . 2010. “Comparative Virulence of *Nosema ceranae* and *Nosema apis* in Individual European Honey Bees.” Veterinary Parasitology 170, no. 3–4: 212–217. 10.1016/j.vetpar.2010.02.010. 20299152

[ece373768-bib-0026] Fries, I. , M.‐P. Chauzat , Y.‐P. Chen , et al. 2013. “Standard Methods for Nosema Research.” Journal of Apicultural Research 52, no. 1: 1–28.

[ece373768-bib-0027] Gaude, E. , M. K. Nakhleh , S. Patassini , et al. 2019. “Targeted Breath Analysis: Exogenous Volatile Organic Compounds (EVOC) as Metabolic Pathway‐Specific Probes.” Journal of Breath Research 13, no. 3: 032001. 10.1088/1752-7163/ab1789. 30965287

[ece373768-bib-0028] Geffre, A. C. , T. Gernat , G. P. Harwood , et al. 2020. “Honey Bee Virus Causes Context‐Dependent Changes in Host Social Behavior.” Proceedings of the National Academy of Sciences of the United States of America 117, no. 19: 10406–10413. 10.1073/pnas.2002268117. 32341145 PMC7229666

[ece373768-bib-0029] Genersch, E. 2010. “American Foulbrood in Honeybees and Its Causative Agent, *Paenibacillus larvae* .” Journal of Invertebrate Pathology 103, no. Suppl 1: S10–S19. 10.1016/j.jip.2009.06.015. 19909971

[ece373768-bib-0030] Genuer, R. , J.‐M. Poggi , C. Tuleau‐Malot , and C. Tuleau . 2010. “Variable Selection Using Random Forests.” Pattern Recognition Letters 31, no. 14: 2225–2236. https://hal.science/hal‐00755489v1.

[ece373768-bib-0031] Gherardi, F. , L. Aquiloni , and E. Tricarico . 2012. “Revisiting Social Recognition Systems in Invertebrates.” Animal Cognition 15, no. 5: 745–762. 10.1007/s10071-012-0513-y. 22639070

[ece373768-bib-0032] Gisder, S. , and E. Genersch . 2017. “Viruses of Commercialized Insect Pollinators.” Journal of Invertebrate Pathology 147: 51–59. 10.1016/j.jip.2016.07.010. 27498219

[ece373768-bib-0033] Goblirsch, M. 2018. “ *Nosema ceranae* Disease of the Honey Bee ( *Apis mellifera* ).” Apidologie 49, no. 1: 131–150.

[ece373768-bib-0034] Goblirsch, M. , Z. Y. Huang , and M. Spivak . 2013. “Physiological and Behavioral Changes in Honey Bees ( *Apis mellifera* ) Induced by *Nosema ceranae* Infection.” PLoS One 8, no. 3: e58165. 10.1371/JOURNAL.PONE.0058165. 23483987 PMC3590174

[ece373768-bib-0035] Gochnauer, T. , and V. Margetts . 1981. “Emission of Volatile Sulphide From Residues of Diseased Honeybee Larvae.” Journal of Apicultural Research 20, no. 2: 110–114.

[ece373768-bib-0036] Gochnauer, T. , and D. Shearer . 1981. “Volatile Acids From Honeybee Larvae Infected With Bacillus Larvae and From a Culture of the Organism.” Journal of Apicultural Research 20, no. 2: 104–109.

[ece373768-bib-0037] Goulson, D. , E. Nicholls , C. Botias , and E. L. Rotheray . 2015. “Bee Declines Driven by Combined Stress From Parasites, Pesticides, and Lack of Flowers.” Science 347, no. 6229: 1255957. 10.1126/science.1255957. 25721506

[ece373768-bib-0038] Higes, M. , R. Martin‐Hernandez , C. Botias , et al. 2008. “How Natural Infection by *Nosema ceranae* Causes Honeybee Colony Collapse.” Environmental Microbiology 10, no. 10: 2659–2669. 10.1111/j.1462-2920.2008.01687.x. 18647336

[ece373768-bib-0039] Higes, M. , R. Martín‐Hernández , and A. Meana . 2010. “ *Nosema ceranae* in Europe: An Emergent Type C Nosemosis.” Apidologie 41, no. 3: 375–392.

[ece373768-bib-0040] Holt, H. L. , and C. M. Grozinger . 2016. “Approaches and Challenges to Managing Nosema (Microspora: Nosematidae) Parasites in Honey Bee (Hymenoptera: Apidae) Colonies.” Journal of Economic Entomology 109, no. 4: 1487–1503. 10.2307/1939574. 27340190

[ece373768-bib-0041] Huang, Q. , and J. D. Evans . 2020. “Targeting the Honey Bee Gut Parasite *Nosema ceranae* With siRNA Positively Affects Gut Bacteria.” BMC Microbiology 20, no. 1: 258. 10.1186/S12866-020-01939-9. 32807095 PMC7433167

[ece373768-bib-0042] Huang, W.‐F. , and L. F. Solter . 2013. “Comparative Development and Tissue Tropism of *Nosema apis* and *Nosema ceranae* .” Journal of Invertebrate Pathology 113, no. 1: 35–41. 23321524 10.1016/j.jip.2013.01.001

[ece373768-bib-0043] Jackson, D. A. 1993. “Stopping Rules in Principal Components Analysis: A Comparison of Heuristical and Statistical Approaches.” Ecology 74, no. 8: 2204–2214.

[ece373768-bib-0044] Kathe, E. , K. Seidelmann , O. Lewkowski , Y. Le Conte , and S. Erler . 2021. “Changes in Chemical Cues of *Melissococcus plutonius* Infected Honey Bee Larvae.” Chemoecology 31, no. 3: 189–200.

[ece373768-bib-0045] Kiesecker, J. M. , D. K. Skelly , K. H. Beard , and E. Preisser . 1999. “Behavioral Reduction of Infection Risk.” Proceedings of the National Academy of Sciences of the United States of America 96, no. 16: 9165–9168. 10.1073/pnas.96.16.9165. 10430913 PMC17750

[ece373768-bib-0046] Kim, J. H. , V. Huang , H. J. Hahn , et al. 2019. “Repurposing Common Food Additives (Benzo Derivatives) as New Anti‐Parasitic Agents.” *Proceedings of 5th International Electronic Conference on Medicinal Chemistry*, 6413. 10.3390/ecmc2019-06413.

[ece373768-bib-0047] Kindt, R. , and R. Coe . 2005. Tree Diversity Analysis. A Manual and Software for Common Statistical Methods for Ecological and Biodiversity Studies. World Agroforestry Centre (ICRAF). http://www.worldagroforestry.org/output/tree‐diversity‐analysis.

[ece373768-bib-0048] Klein, A. M. , B. E. Vaissiere , J. H. Cane , et al. 2007. “Importance of Pollinators in Changing Landscapes for World Crops.” Proceedings of the Royal Society B: Biological Sciences 274, no. 1608: 303–313. 10.1098/rspb.2006.3721. PMC1702377 17164193

[ece373768-bib-0049] Lattorff, H. M. G. 2022. “Increased Stress Levels in Caged Honeybee ( *Apis mellifera* ) (hymenoptera: Apidae) Workers.” Stress 2, no. 4: 373–383.

[ece373768-bib-0050] Lau, E. , J. Maccaro , Q. S. McFrederick , and J. C. Nieh . 2024. “Exploring the Interactions Between *Nosema ceranae* Infection and the Honey Bee Gut Microbiome.” Scientific Reports 14, no. 1: 20037. 10.1038/s41598-024-67796-y. 39198535 PMC11358482

[ece373768-bib-0051] Lee, S. , S. Lim , Y. S. Choi , M. L. Lee , and H. W. Kwon . 2020. “Volatile Disease Markers of American Foulbrood‐Infected Larvae in *Apis mellifera* .” Journal of Insect Physiology 122: 104040. 10.1016/j.jinsphys.2020.104040. 32126215

[ece373768-bib-0052] Legendre, P. , and L. Legendre . 2012. Numerical Ecology. Vol. 24. Elsevier.

[ece373768-bib-0053] Li, Y.‐H. , Z.‐T. Chang , M.‐R. Yen , et al. 2022. “Transcriptome of *Nosema ceranae* and Upregulated Microsporidia Genes During Its Infection of Western Honey Bee ( *Apis mellifera* ).” Insects 13, no. 8: 716. 36005340 10.3390/insects13080716 PMC9409478

[ece373768-bib-0054] Liaw, A. , and M. Wiener . 2002. “Classification and Regression by RandomForest.” R News 2, no. 3: 18–22. https://CRAN.R‐project.org/doc/Rnews/.

[ece373768-bib-0055] Liberles, S. D. 2014. “Mammalian Pheromones.” Annual Review of Physiology 76, no. 1: 151–175. 10.1146/annurev-physiol-021113-170334 PMC4310675 23988175

[ece373768-bib-0056] Liendo, M. C. , I. Muntaabski , R. M. Russo , et al. 2021. “Temporal Changes in Volatile Profiles of *Varroa destructor* ‐Infested Brood May Trigger Hygienic Behavior in *Apis mellifera* .” Entomologia Experimentalis et Applicata 169, no. 6: 563–574. 10.1111/eea.13048.

[ece373768-bib-0057] Light, M. , D. Shutler , G. C. Cutler , and N. K. Hillier . 2020. “ *Varroa destructor* Mite Electrophysiological Responses to Honey Bee ( *Apis mellifera* ) Colony Volatiles.” Experimental and Applied Acarology 81, no. 4: 495–514. 32700265 10.1007/s10493-020-00519-w

[ece373768-bib-0058] Liolios, V. , D. Kanelis , C. Tananaki , and M.‐A. Rodopoulou . 2022. “A Comparative Study of Healthy and American Foulbrood‐Infected Bee Brood ( *Apis mellifera* L.) Through the Investigation of Volatile Compounds.” Agriculture 12, no. 6: 812.

[ece373768-bib-0059] Liu, J. , R. Zhang , R. Tang , et al. 2022. “The Role of Honey Bee Derived Aliphatic Esters in the Host‐Finding Behavior of *Varroa destructor* .” Insects 14, no. 1: 24. 36661952 10.3390/insects14010024 PMC9863403

[ece373768-bib-0060] Madhwal, S. , M. Shin , A. Kapoor , et al. 2020. “Metabolic Control of Cellular Immune‐Competency by Odors in Drosophila.” eLife 9: e60376. 33372660 10.7554/eLife.60376 PMC7808736

[ece373768-bib-0061] Martín‐Hernández, R. , C. Bartolomé , N. Chejanovsky , et al. 2018. “ *Nosema ceranae* in *Apis mellifera* : A 12 Years Postdetection Perspective.” Environmental Microbiology 20, no. 4: 1302–1329. 29575513 10.1111/1462-2920.14103

[ece373768-bib-0062] McDonnell, C. M. , C. Alaux , H. Parrinello , et al. 2013. “Ecto‐ and Endoparasite Induce Similar Chemical and Brain Neurogenomic Responses in the Honey Bee ( *Apis mellifera* ).” BMC Ecology 13, no. 1: 25. 10.1186/1472-6785-13-25. 23866001 PMC3725162

[ece373768-bib-0063] Mulholland, G. E. , B. E. Traver , N. G. Johnson , and R. D. Fell . 2012. “Individual Variability of *Nosema ceranae* Infections in *Apis mellifera* Colonies.” Insects 3, no. 4: 1143–1155. 26466731 10.3390/insects3041143 PMC4553568

[ece373768-bib-0064] Murray, Z. L. , R. A. Keyzers , R. F. Barbieri , A. P. Digby , and P. J. Lester . 2015. “Two Pathogens Change Cuticular Hydrocarbon Profiles but Neither Elicit a Social Behavioural Change in Infected Honey Bees, *Apis mellifera* (Apidae: Hymenoptera).” Austral Entomology 55, no. 2: 147–153. 10.1111/aen.12165.

[ece373768-bib-0065] National Institute of Standards and Technology . 2020. NIST Mass Spectral Library (NIST 20). NIST.

[ece373768-bib-0066] Nazzi, F. , G. Della Vedova , and M. d'Agaro . 2004. “A Semiochemical From Brood Cells Infested by *Varroa destructor* Triggers Hygienic Behaviour in *Apis mellifera* .” Apidologie 35, no. 1: 65–70.

[ece373768-bib-0067] Nazzi, F. , N. Milani , and G. Della Vedova . 2002. “(Z)‐8‐Heptadecene From Infested Cells Reduces the Reproduction of *Varroa destructor* Under Laboratory Conditions.” Journal of Chemical Ecology 28, no. 11: 2181–2190. 10.1023/A:1021041130593. 12523561

[ece373768-bib-0068] Noël, A. , C. Dumas , E. Rottier , et al. 2025. “Identification of Five Volatile Organic Compounds That Trigger Hygienic and Recapping Behaviours in the Honey Bee ( *Apis mellifera* ).” International Journal for Parasitology 55: 351–363. 39900171 10.1016/j.ijpara.2025.01.009

[ece373768-bib-0069] Oksanen, J. , G. L. Simpson , F. G. Blanchet , et al. 2025. “vegan: Community Ecology Package.” R Package Version 2.8‐0 ed.

[ece373768-bib-0070] Ollerton, J. 2017. “Pollinator Diversity: Distribution, Ecological Function, and Conservation.” Annual Review of Ecology, Evolution, and Systematics 48, no. 1: 353–376.

[ece373768-bib-0071] Pasca, C. , L. A. Marghitaș , C. Șonea , O. Bobiș , I. A. Buzura‐Matei , and D. S. Dezmirean . 2019. “A Review of *Nosema cerane* and *Nosema apis*: Caracterization and Impact for Beekeeping.” Bulletin of the University of Agricultural Sciences & Veterinary Medicine Cluj‐Napoca. Animal Science & Biotechnologies 76, no. 2: 77–87. 10.15835/buasvmcn-asb:0018.19.

[ece373768-bib-0072] Paxton, R. J. , J. Klee , S. Korpela , and I. Fries . 2007. “ *Nosema ceranae* Has Infected *Apis mellifera* in Europe Since at Least 1998 and May Be More Virulent Than *Nosema apis* .” Apidologie 38, no. 6: 558–565.

[ece373768-bib-0073] Peled, N. , R. Ionescu , P. Nol , et al. 2012. “Detection of Volatile Organic Compounds in Cattle Naturally Infected With *Mycobacterium bovis* .” Sensors and Actuators B: Chemical 171: 588–594. 10.1016/j.snb.2012.05.038.

[ece373768-bib-0074] Potts, S. G. , S. P. Roberts , R. Dean , et al. 2010. “Declines of Managed Honey Bees and Beekeepers in Europe.” Journal of Apicultural Research 49, no. 1: 15–22.

[ece373768-bib-0075] R Core Team . 2023. A Language and Environment for Statistical Computing. R Foundation for Statistical Computing. https://www.R‐project.org/.

[ece373768-bib-0076] Reilly, J. , I. Bartomeus , D. Simpson , A. Allen‐Perkins , L. Garibaldi , and R. Winfree . 2024. “Wild Insects and Honey Bees Are Equally Important to Crop Yields in a Global Analysis.” Global Ecology and Biogeography 33, no. 7: e13843.

[ece373768-bib-0077] Rubanov, A. , K. A. Russell , J. A. Rothman , J. C. Nieh , and Q. S. McFrederick . 2019. “Intensity of *Nosema ceranae* Infection Is Associated With Specific Honey Bee Gut Bacteria and Weakly Associated With Gut Microbiome Structure.” Scientific Reports 9, no. 1: 3820. 10.1038/s41598-019-40347-6. 30846803 PMC6405881

[ece373768-bib-0078] Schöning, C. , S. Gisder , S. Geiselhardt , et al. 2012. “Evidence for Damage‐Dependent Hygienic Behaviour Towards *Varroa destructor* ‐Parasitised Brood in the Western Honey Bee, *Apis mellifera* .” Journal of Experimental Biology 215, no. Pt 2: 264–271. 10.1242/jeb.062562. 22189770

[ece373768-bib-0079] Schwarz, R. S. , N. A. Moran , and J. D. Evans . 2016. “Early Gut Colonizers Shape Parasite Susceptibility and Microbiota Composition in Honey Bee Workers.” Proceedings of the National Academy of Sciences of the United States of America 113, no. 33: 9345–9350. 10.1073/pnas.1606631113. 27482088 PMC4995961

[ece373768-bib-0080] Shirasu, M. , and K. Touhara . 2011. “The Scent of Disease: Volatile Organic Compounds of the Human Body Related to Disease and Disorder.” Journal of Biochemistry 150, no. 3: 257–266. 10.1093/jb/mvr090. 21771869

[ece373768-bib-0081] Simone‐Finstrom, M. , R. S. Borba , M. Wilson , and M. Spivak . 2017. “Propolis Counteracts Some Threats to Honey Bee Health.” Insects 8, no. 2: 46. 28468244 10.3390/insects8020046 PMC5492060

[ece373768-bib-0082] Simone‐Finstrom, M. D. , and M. Spivak . 2012. “Increased Resin Collection After Parasite Challenge: A Case of Self‐Medication in Honey Bees?” PLoS One 7, no. 3: e34601. 22479650 10.1371/journal.pone.0034601 PMC3315539

[ece373768-bib-0083] Smith, G. , and J. Bromenshenk . 2002. “Volatile and Semi‐Volatile Organic Compounds in Beehive Atmospheres.” In Honey Bees, edited by G. C. Smith , G. Alnasser , D. C. Jones , and J. Bromenshenk , 12–41. CRC Press.

[ece373768-bib-0084] Swanson, J. A. , B. Torto , S. A. Kells , K. A. Mesce , J. H. Tumlinson , and M. Spivak . 2009. “Odorants That Induce Hygienic Behavior in Honeybees: Identification of Volatile Compounds in Chalkbrood‐Infected Honeybee Larvae.” Journal of Chemical Ecology 35, no. 9: 1108–1116. 10.1007/s10886-009-9683-8. 19816752

[ece373768-bib-0085] Tibbetts, E. A. , and J. Dale . 2007. “Individual Recognition: It Is Good to Be Different.” Trends in Ecology & Evolution 22, no. 10: 529–537. 17904686 10.1016/j.tree.2007.09.001

[ece373768-bib-0086] Traynor, K. S. , F. Mondet , J. R. de Miranda , et al. 2020. “ *Varroa destructor* : A Complex Parasite, Crippling Honey Bees Worldwide.” Trends in Parasitology 36, no. 7: 592–606. 32456963 10.1016/j.pt.2020.04.004

[ece373768-bib-0087] Wagoner, K. , M. Spivak , A. Hefetz , T. Reams , and O. Rueppell . 2019. “Stock‐Specific Chemical Brood Signals Are Induced by Varroa and Deformed Wing Virus, and Elicit Hygienic Response in the Honey Bee.” Scientific Reports 9, no. 1: 8753. 10.1038/s41598-019-45008-2. 31217481 PMC6584651

[ece373768-bib-0088] Walker, T. N. , and W. O. Hughes . 2009. “Adaptive Social Immunity in Leaf‐Cutting Ants.” Biology Letters 5, no. 4: 446–448. 19411266 10.1098/rsbl.2009.0107 PMC2781909

[ece373768-bib-0089] Wickham, H. 2011. “ggplot2.” Wiley Interdisciplinary Reviews: Computational Statistics 3, no. 2: 180–185.

[ece373768-bib-0090] Wyatt, T. D. 2014. Pheromones and Animal Behavior: Chemical Signals and Signatures. Cambridge University Press.

[ece373768-bib-0091] Zhao, Q. , X. Wang , A. Mustafa , et al. 2025. “Varroa Volatiles Offer Chemical Cues to Honey Bees for Initial Parasitic Recognition.” Biomolecules 15, no. 1: 66. 10.3390/biom15010066. 39858461 PMC11764367

