# Supplementary material for: Wake Up and Smell the Infected Bees: Volatile Cues of Vairimorpha Infection in Honey Bees
Source: Ecol Evol. 2026 Jun 4;16(6):e73768. doi: 10.1002/ece3.73768 (PMC13238872; doi:10.1002/ece3.73768)
Supplement: Supplementary file 1 — Figure S1: Custom headspace sampling bags consisting of a hoarding cage, 50% w/v sucrose feeder, and Eppendorf sampling port connected to the interior of the cage. 3 L of headspace was extracted onto thermal desorption tubes using an ACTI‐VOC pump at 200 mL/min. Figure S2: Top 30 volatile organic compounds (VOCs) ranked by their importance to classification accuracy in the random forest model based on all 71 VOCs. Higher values indicate a greater contribution to model performance. The dashed red line indicates the cut‐off point used to select the most important compounds for discriminating between combined treatment and days post‐infection (dpi) variables, defined by the largest step change in mean decrease accuracy. Figure S3: Top 30 volatile organic compounds (VOCs) ranked by their contribution to the redundancy analysis (RDA) model based on all 71 VOCs. Higher magnitudes indicate a stronger contribution to constrained variance. The dashed red line indicates the cut‐off point used to select the most important compounds for discriminating between combined treatment and days post‐infection (dpi) variables. Figure S4: Infection dynamics of Vairimorpha spp. in reference and experimental bees. Reference bees were destructively sampled at 0, 3, 6, 9, 12, and 14 days post‐infection (dpi) to confirm infection progression, while experimental bees used for volatile organic compound sampling were only sampled at 14 dpi. Each point represents a pooled sample of three bees. The black line and shaded ribbon show a loess smoother with ±95% confidence interval fitted across all data. Due to natural mortality, only one reference cage replicate was available at 12 and 14 dpi. Spore loads rose from 6.0 ± 2.8 × 10^4^ at 3 dpi to 2.0 ± 0.27 × 10^6^ at 6 dpi reaching 2.7 ± 0.18 × 10^7^ at 9 dpi, and exceeded 108 spores per bee by 12 dpi. At 14 dpi, both reference and experimental bees exhibited similarly high spore loads (1.18 ± 0.14 × 10^8^). Figure S5: Number of bees alive per cage across th [file ECE3-16-e73768-s001.docx]

## Appendix


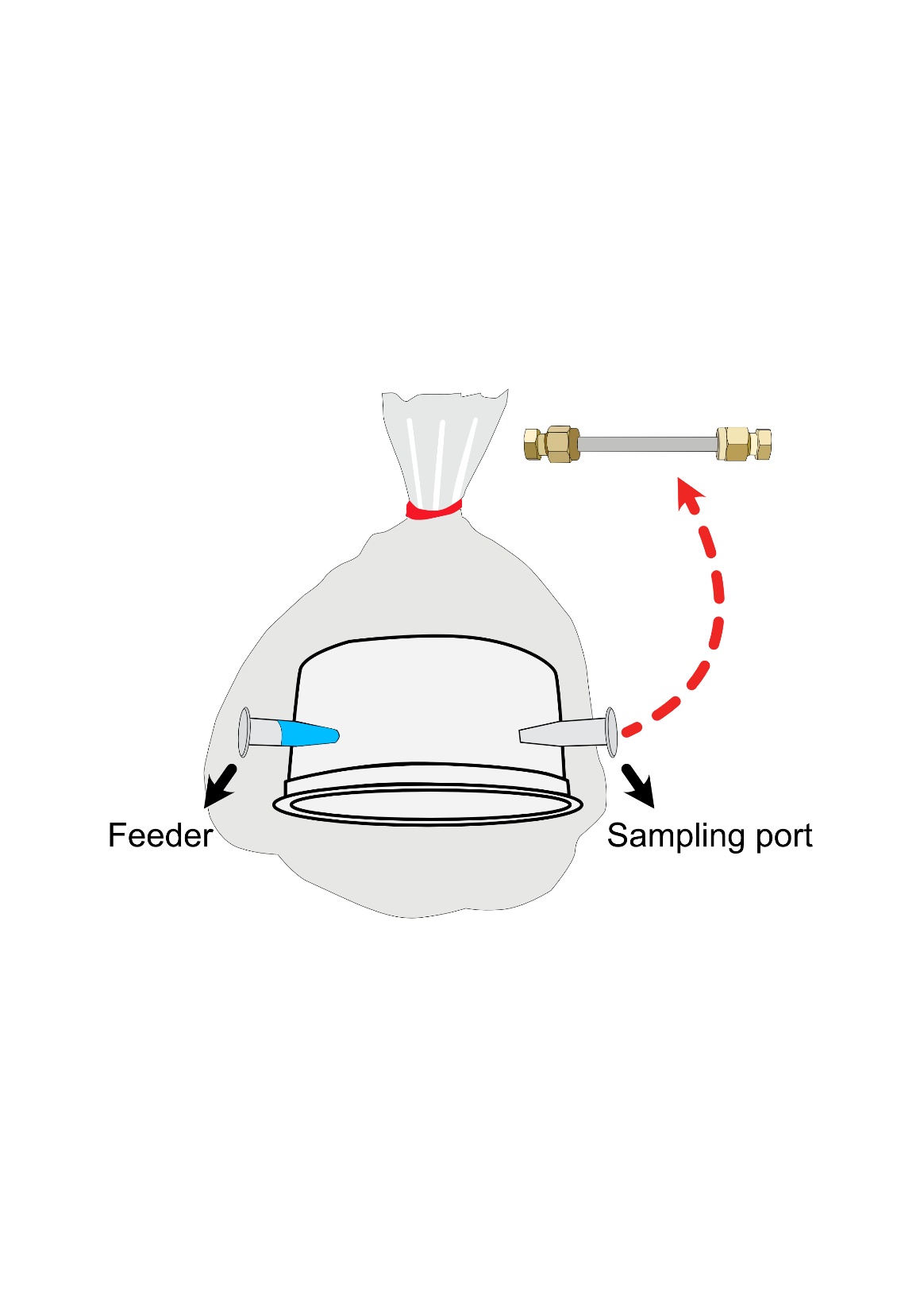


**Figure** **S1: Custom headspace sampling bags consisting of a hoarding cage, 50% w/v sucrose feeder, and Eppendorf sampling port connected to the interior of the cage.**

3 L of headspace was extracted onto thermal desorption tubes using an ACTI-VOC pump at 200 mL/min.


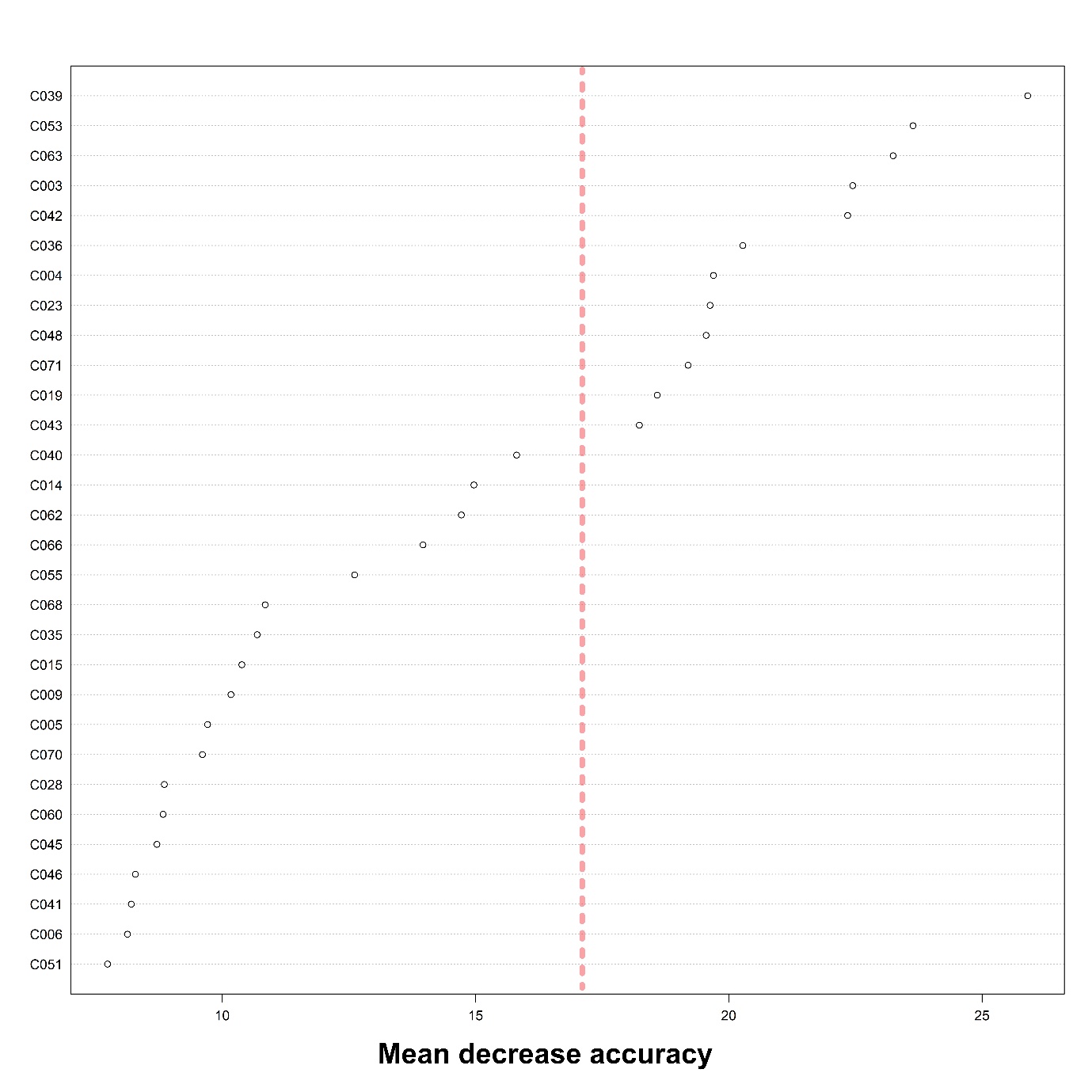
**Figure S2: Top 30 volatile organic compounds (VOCs) ranked by their importance to classification accuracy in the random forest model based on all 71 VOCs.** Higher values indicate a greater contribution to model performance. The dashed red line indicates the cut-off point used to select the most important compounds for discriminating between combined treatment and days post-infection (dpi) variables, defined by the largest step change in mean decrease accuracy.


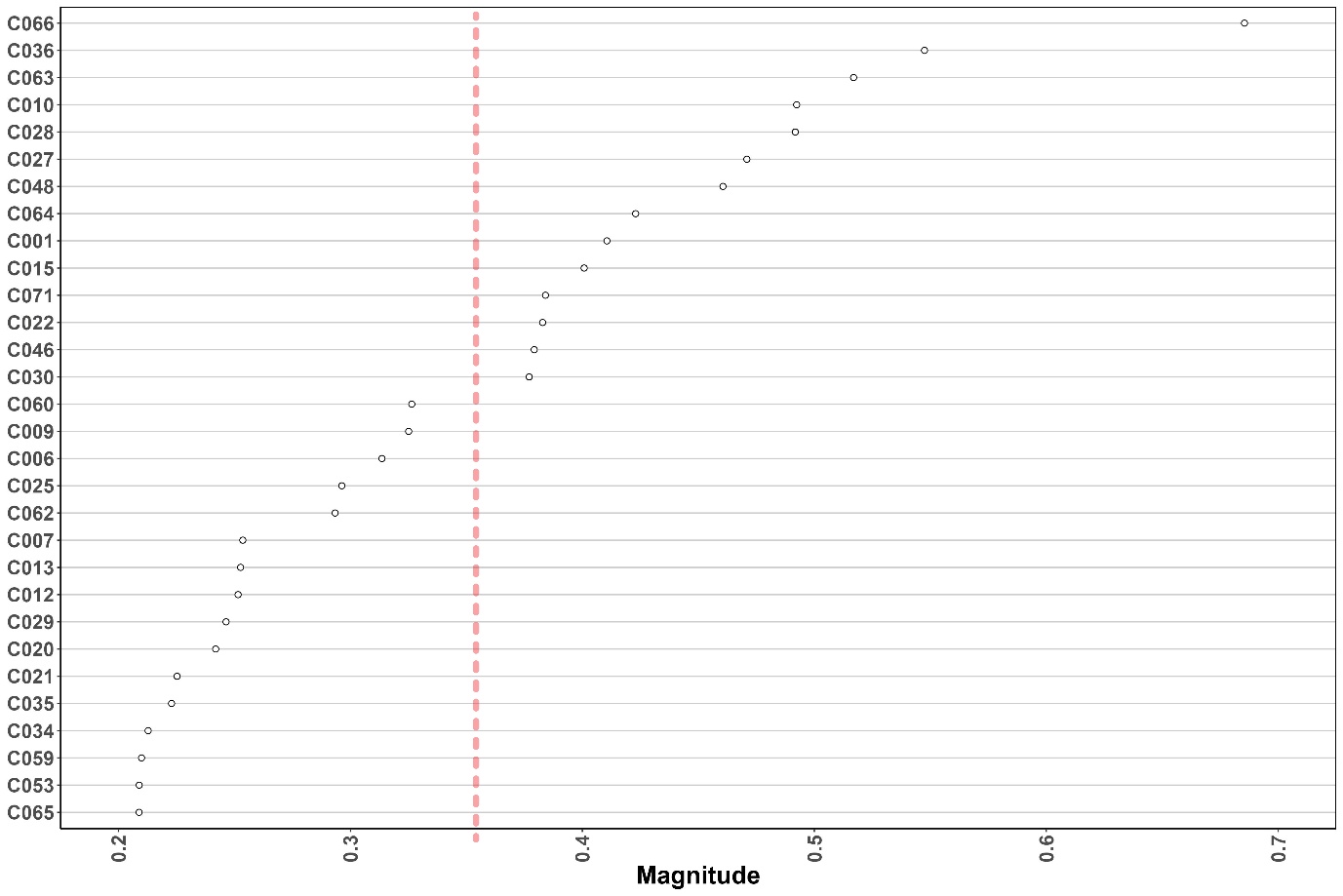


**Figure S3: Top 30 volatile organic compounds (VOCs) ranked by their contribution to the redundancy analysis (RDA) model based on all 71 VOCs.** Higher magnitudes indicate a stronger contribution to constrained variance. The dashed red line indicates the cut-off point used to select the most important compounds for discriminating between combined treatment and days post-infection (dpi) variables.


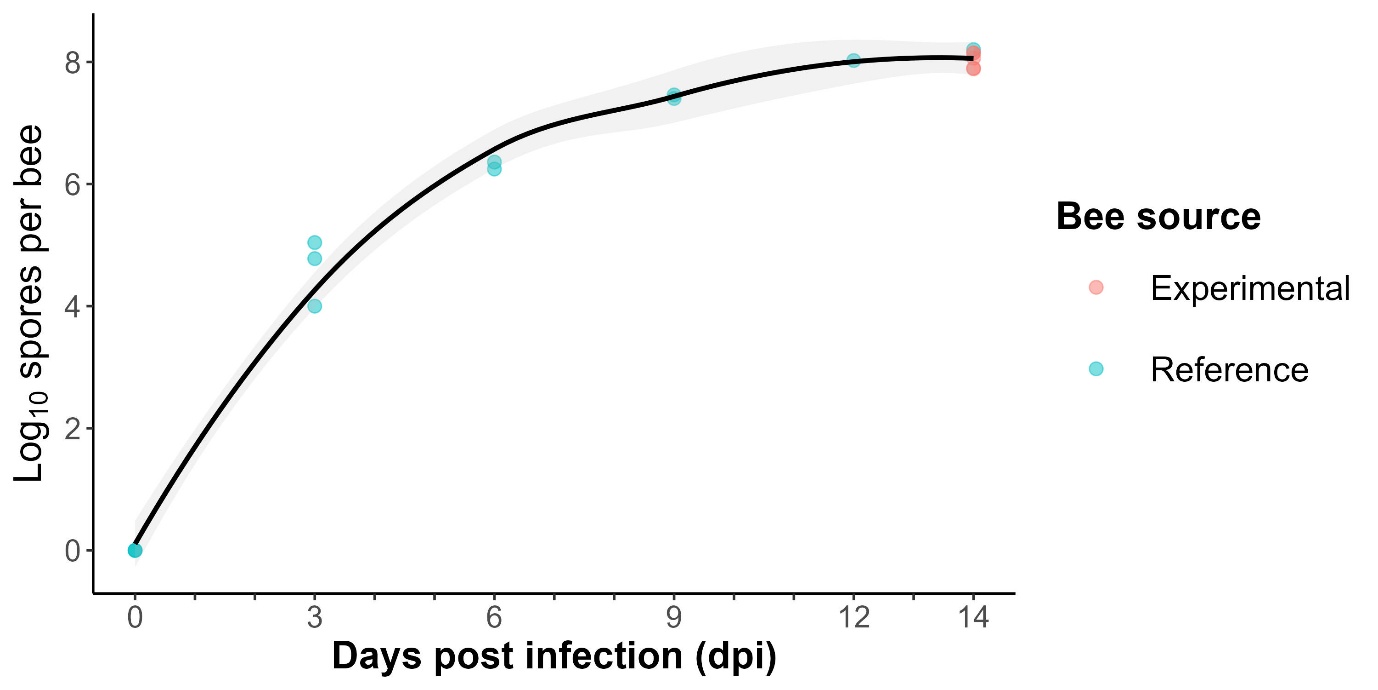


**Figure S4: Infection dynamics of *Vairimorpha* spp. in reference and experimental bees.** Reference bees were destructively sampled at 0, 3, 6, 9, 12, and 14 days post-infection (dpi) to confirm infection progression, while experimental bees used for volatile organic compound sampling were only sampled at 14 dpi. Each point represents a pooled sample of three bees. The black line and shaded ribbon show a loess smoother with ±95% confidence interval fitted across all data. Due to natural mortality, only one reference cage replicate was available at 12 and 14 dpi. Spore loads rose from 6.0 ± 2.8_×_10^4^ at 3 dpi to 2.0 ± 0.27_×_10^6^ at 6 dpi reaching 2.7 ± 0.18_×_10^7^ at 9 dpi, and exceeded 10^8^ spores per bee by 12 dpi. At 14 dpi, both reference and experimental bees exhibited similarly high spore loads (1.18 ± 0.14_×_10^8^)

**
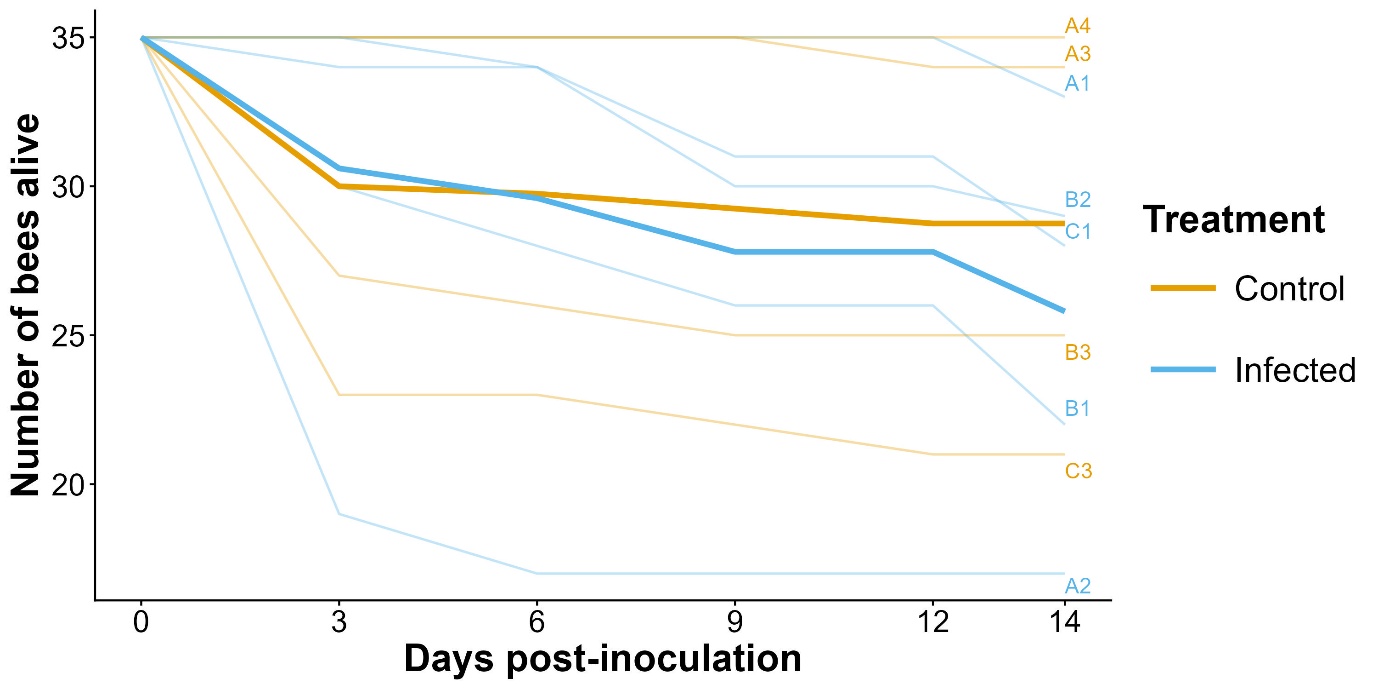
Figure S5: Number of bees alive per cage across the 14-day experiment for control and infected treatments.** Thin lines represent individual cages (labels shown at day 14); thick lines show treatment means. Mortality declined over time in both treatments, with similar trajectories despite variation among cages.

**Table S1: The 71 volatile organic compounds (VOCs) identified in infected and uninfected bees across all timepoints. Retention indices (RI) were calculated from experimental data and compared against literature values from the NIST 2020 mass spectral library. Where multiple database entries were available, the experimental RI value for non-standard non-polar columns was adopted for consistency throughout, although column-specific values were available in some cases.**

| **Compound** | **Component** | **CAS number** | **RI (exp)** | **RI (lit)** | **Family** |
| --- | --- | --- | --- | --- | --- |
| 1-Octanol‚ 2‚2-dimethyl- | C001 | 2370-14-1 | 1094 | n.f. | alcohol |
| 1-Octene‚ 2‚6-dimethyl- | C002 | 6874-29-9 | 946 | 954 | alkene |
| 1‚3-Cyclopentadiene‚ 1‚2‚3‚4-tetramethyl-5-methylene- | C003 | 76089-59-3 | 1167 | n.f. | cycloalkene |
| 1H-Indene‚ 2‚3-dihydro-4-methyl- | C004 | 824-22-6 | 1157 | 1141 | aromatic hydrocarbon |
| 1H-Indene‚ 2‚3-dihydro-5-methyl- | C005 | 874-35-1 | 1166 | 1135 | aromatic hydrocarbon |
| 2‚3-Dimethyl-2-heptene | C006 | 3074-64-4 | 858 | 878 | alkene |
| 2‚4-Di-tert-butylphenol | C007 | 96-76-4 | 1518 | 1514 | phenol |
| 2‚4-Dimethyl-1-heptene | C008 | 19549-87-2 | 864 | 855 | alkene |
| 2‚6-Dimethyldecane | C009 | 13150-81-7 | 1106 | 1112 | alkane |
| 3-Ethyl-3-methylheptane | C010 | 17302-01-1 | 1015 | 953 | alkane |
| 3‚4-Dimethylcumene | C011 | 4132-77-8 | 1208 | n.f. | alkylbenzene |
| Alkane02 | C012 | N.D. | 1020 |  | alkane |
| Alkane03 | C013 | N.D. | 1055 |  | alkane |
| Alkane04 | C014 | N.D. | 1056 |  | alkane |
| Alkane07 | C015 | N.D. | 1078 |  | alkane |
| Alkane13 | C016 | N.D. | 1194 |  | alkane |
| Alkane14 | C017 | N.D. | 1198 |  | alkane |
| Alkane15 | C018 | N.D. | 1208 |  | alkane |
| Alkane17 | C019 | N.D. | 1230 |  | alkane |
| Alkane18 | C020 | N.D. | 1239 |  | alkane |
| Alkane19 | C021 | N.D. | 1245 |  | alkane |
| Alkane21 | C022 | N.D. | 1260 |  | alkane |
| Alkane22 | C023 | N.D. | 1272 |  | alkane |
| Alkane23 | C024 | N.D. | 1280 |  | alkane |
| Alkane24 | C025 | N.D. | 1293 |  | alkane |
| Alkane25 | C026 | N.D. | 1318 |  | alkane |
| Alkane26 | C027 | N.D. | 1328 |  | alkane |
| Alkane27 | C028 | N.D. | 1337 |  | alkane |
| Alkane28 | C029 | N.D. | 1347 |  | alkane |
| Alkane30 | C030 | N.D. | 1528 |  | alkane |
| Alkanol02 | C031 | N.D. | 1310 |  | alkanol |
| Alkanol03 | C032 | N.D. | 1301 |  | alkanol |
| Alkene09 | C033 | N.D. | 1259 |  | alkene |
| Alkene18 | C034 | N.D. | 1307 |  | alkene |
| Alkene19 | C035 | N.D. | 1316 |  | alkene |
| Benzaldehyde‚ 3‚4-dimethyl- | C036 | 5973-71-7 | 1239 | 1236 | aldehyde |
| Benzene‚ 1-ethyl-3‚5-dimethyl- | C037 | 934-74-7 | 1070 | 1059 | aromatic hydrocarbon |
| Benzene‚ 1-methyl-3-(1-methylethyl)- | C038 | 535-77-3 | 1039 | 1022 | aromatic hydrocarbon |
| Benzene‚ 1‚2‚3‚4-tetramethyl- | C039 | 488-23-3 | 1134 | 1143 | aromatic hydrocarbon |
| Benzene‚ 1‚2‚3‚5-tetramethyl- | C040 | 527-53-7 | 1130 | 1117 | aromatic hydrocarbon |
| Benzene‚ 1‚2‚4-trimethyl- | C041 | 95-63-6 | 1011 | 990 | aromatic hydrocarbon |
| Benzene‚ 1‚3-bis(1‚1-dimethylethyl)- | C042 | 1014-60-4 | 1255 | 1249 | aromatic hydrocarbon |
| Benzene‚ 2-ethyl-1‚3-dimethyl- | C043 | 2870-04-4 | 1097 | 1087 | aromatic hydrocarbon |
| C3-Benzene01 | C044 | N.D. | 1038 | n/a | aromatic hydrocarbon |
| Cyclohexane‚ 1‚2‚3-trimethyl-‚ (1α‚2α‚3β)- | C045 | 7667-55-2 | 910 | 908 | cycloalkane |
| Decane | C046 | 124-18-5 | 1000 | 1000 | alkane |
| Decane‚ 2-methyl- | C047 | 6975-98-0 | 1062 | 1064 | alkane |
| Dodecane | C048 | 112-40-3 | 1200 | 1200 | alkane |
| Ethylbenzene | C049 | 100-41-4 | 889 | 855 | aromatic hydrocarbon |
| Heptadecane | C050 | 629-78-7 | 1700 | 1700 | alkane |
| Heptane‚ 2‚4-dimethyl- | C051 | 2213-23-2 | 832 | 821 | alkane |
| Hexane‚ 2‚3‚3-trimethyl- | C052 | 16747-28-7 | 847 | 840 | alkane |
| Naphthalene | C053 | 91-20-3 | 1210 | 1182 | aromatic hydrocarbon |
| Naphthalene‚ 1-methyl- | C054 | 90-12-0 | 1322 | 1307 | aromatic hydrocarbon |
| Nonanal | C055 | 124-19-6 | 1115 | 1104 | aldehyde |
| Nonane‚ 2-methyl- | C056 | 871-83-0 | 972 | 964 | alkane |
| Nonane‚ 2‚5-dimethyl- | C057 | 17302-27-1 | 1022 | 1021 | alkane |
| o-Xylene | C058 | 95-47-6 | 917 | 888 | aromatic hydrocarbon |
| p-Xylene | C059 | 106-42-3 | 897 | 865 | aromatic hydrocarbon |
| Pentadecane | C060 | 629-62-9 | 1500 | 1500 | alkane |
| Styrene | C061 | 100-42-5 | 917 | 893 | aromatic hydrocarbon |
| Terpene01 | C062 | N.D. | 939 | n/a | terpenoid |
| Tetradecane | C063 | 629-59-4 | 1400 | 1400 | alkane |
| Toluene | C064 | 108-88-3 | 816 | 763 | aromatic hydrocarbon |
| Tridecane | C065 | 629-50-5 | 1300 | 1300 | alkane |
| Undecane | C066 | 1120-21-4 | 1100 | 1100 | alkane |
| Undecane‚ 2-methyl- | C067 | 7045-71-8 | 1164 | 1164 | alkane |
| Undecane‚ 2‚6-dimethyl- | C068 | 17301-23-4 | 1212 | 1210 | alkane |
| α-Pinene | C069 | 80-56-8 | 950 | 937 | terpenoid |
| 1-Octanol‚ Dimethyl- | C070 | N.D. | 1099 | n/a | alcohol |
| Unknown#1 | C071 | N.D. | 1083 | n/a | N.D. |

**Table S2: Classification success (%) from canonical analysis of principal coordinates (CAP) using all 71 VOCs across treatments and days post-infection.**

CAP achieved an overall classification success of 46%.

| **Days post-infection** | **Treatment** | **Classification success (%)** |
| --- | --- | --- |
| 0 | Control | 75 |
| 0 | Infected | 0 |
| 3 | Control | 25 |
| 3 | Infected | 80 |
| 6 | Control | 50 |
| 6 | Infected | 33 |
| 9 | Control | 50 |
| 9 | Infected | 60 |
| 12 | Control | 100 |
| 12 | Infected | 80 |
| 14 | Control | 0 |
| 14 | Infected | 0 |

**Table S3: Classification success (%) from canonical analysis of principal components (CAP) using the top 12 VOCs identified as most important in the random forest model across treatments and days post-infection.**

CAP achieved an overall classification success of 67%.

| **Days post-infection** | **Treatment** | **Classification success (%)** |
| --- | --- | --- |
| 0 | Control | 75 |
| 0 | Infected | 60 |
| 3 | Control | 25 |
| 3 | Infected | 40 |
| 6 | Control | 75 |
| 6 | Infected | 100 |
| 9 | Control | 25 |
| 9 | Infected | 80 |
| 12 | Control | 100 |
| 12 | Infected | 100 |
| 14 | Control | 50 |
| 14 | Infected | 80 |
